# Lignin intermediates lead to phenyl acid formation and microbial community shifts in meso- and thermophilic batch reactors

**DOI:** 10.1186/s13068-020-01855-0

**Published:** 2021-01-20

**Authors:** Eva Maria Prem, Mira Mutschlechner, Blaz Stres, Paul Illmer, Andreas Otto Wagner

**Affiliations:** 1 Department of Microbiology, Universität Innsbruck, Technikerstraße 25d, 6020 Innsbruck, Austria; 2 Department of Animal Science, Biotechnical Faculty, University of Ljubljana, Jamnikarjeva 101, 1000 Ljubljana, Slovenia; 3 Institute of Sanitary Engineering, Faculty of Civil and Geodetic Engineering, University of Ljubljana, Jamova 2, 1000 Ljubljana, Slovenia; 4 Department of Automation, Biocybernetics and Robotics, Jozef Štefan Institute, Jamova 39, 1000 Ljubljana, Slovenia

**Keywords:** Bio-methane, Phenyl acids, Anaerobic digestion, Lignin intermediates, Amplicon sequencing

## Abstract

**Background:**

Lignin intermediates resulting from lignocellulose degradation have been suspected to hinder anaerobic mineralisation of organic materials to biogas. Phenyl acids like phenylacetate (PAA) are early detectable intermediates during anaerobic digestion (AD) of aromatic compounds. Studying the phenyl acid formation dynamics and concomitant microbial community shifts can help to understand the microbial interdependencies during AD of aromatic compounds and may be beneficial to counteract disturbances.

**Results:**

The length of the aliphatic side chain and chemical structure of the benzene side group(s) had an influence on the methanogenic system. PAA, phenylpropionate (PPA), and phenylbutyrate (PBA) accumulations showed that the respective lignin intermediate was degraded but that there were metabolic restrictions as the phenyl acids were not effectively processed. Metagenomic analyses confirmed that mesophilic genera like *Fastidiosipila* or *Syntrophomonas* and thermophilic genera like *Lactobacillus*,* Bacillus*, *Geobacillus*, and *Tissierella* are associated with phenyl acid formation. Acetoclastic methanogenesis was prevalent in mesophilic samples at low and medium overload conditions, whereas *Methanoculleus* spp. dominated at high overload conditions when methane production was restricted. In medium carbon load reactors under thermophilic conditions, syntrophic acetate oxidation (SAO)-induced hydrogenotrophic methanogenesis was the most important process despite the fact that acetoclastic methanogenesis would thermodynamically be more favourable. As acetoclastic methanogens were restricted at medium and high overload conditions, syntrophic acetate oxidising bacteria and their hydrogenotrophic partners could step in for acetate consumption.

**Conclusions:**

PAA, PPA, and PBA were early indicators for upcoming process failures. Acetoclastic methanogens were one of the first microorganisms to be impaired by aromatic compounds, and shifts to syntrophic acetate oxidation coupled to hydrogenotrophic methanogenesis occurred in thermophilic reactors. Previously assumed associations of specific meso- and thermophilic genera with anaerobic phenyl acid formation could be confirmed.

## Background

Bio-methane is considered a valuable, carbon–neutral energy source, which is generated by gradual degradation of complex organic substrates under anaerobic conditions [1]. The fact that methane as end product of anaerobic digestion (AD) can still be used for energy exploitation shows that degradation processes under anoxic conditions [2, 3] yield far less energy for the microorganisms than under aerobic conditions. As a consequence, a variety of anaerobic microorganisms depend on each other to overcome thermodynamic restrictions [4]. These microbial interdependencies, especially the obligatory mutualistic (syntrophic) co-operations, are still not sufficiently understood despite their significance for maintaining anaerobic systems [5]. The (final) degradation step is mainly done by hydrogenotrophic (H_2_/CO_2_) or acetoclastic (acetate) methanogens, which enable thermodynamic efficiency by removal of excess reducing equivalents. A low methanogenic activity can restrict upstream degradation steps as metabolic intermediates accumulate and reactions become endergonic [5].

The use of locally collected, organic (waste) materials, which are not potential food sources, is highly desirable in terms of an effective, sustainable, and ethically acceptable energy management [6]. Over the last decades, chemical, physical, and biological pre-treatment strategies empowered the use of rather recalcitrant or otherwise unsuitable organic wastes like lignocellulose or proteins for biogas formation [7]. However, these new techniques also led to an increasing input of potentially problematic compounds [8] like hydrogen sulphide, anti-biotics [9], or ammonium [10, 11]. Another underexplored group of potential inhibitors are (monocyclic) aromatic substances [12–14]. Despite their ubiquitous and abundant occurrence, the degradation of the nonpolar, six-carbon ring structures (benzenes) is considered challenging, especially under anaerobic conditions [15]. Apart from some exceptions in the animal kingdom, prokaryotes and fungi are considered to be the only life forms capable of completely degrading benzene rings [16]. Hence, the accumulation of aromatic compounds is an ever-present threat in anoxic environments, especially under substrate overload conditions [12, 17]. One group of monocyclic aromatic compounds are phenyl acids that include phenylacetate (PAA), phenylpropionate (PPA), or phenylbutyrate (PBA). Studies on PPA [18] and especially on PBA [19] are very rare, even though they represent relevant intermediates in the AD cascade prior to the anaerobic ring cleavage. A cascade-like increase/decrease from PAA to PPA to PBA could be observed when straw from grain was anaerobically degraded under overload conditions. It was concluded that an increase in PAA concentration was an early indicator for overload conditions, whereas an increase in PBA concentration indicated a switch from using easier degradable substrates to more recalcitrant (lignocellulosic) materials [19].

Previous studies also indicated that fermenting bacteria in general and the microbial phyla *Firmicutes* and *Proteobacteria* in particular are responsible for aromatic compound dynamics during AD [18, 20]. More recently, genera like *Acetomicrobium* spp., *Sedimentibacter* spp., *Tepidanaerobacter* spp., or *Sporanaerobacter* spp. were shown to be important biomarkers for high phenyl acid concentrations in batch reactors fed with aromatic amino acids [21]. Whether these genera are directly or indirectly associated with phenyl acid formation remains to be elucidated. The thermodynamically difficult cleavage of the benzene ring itself seemed to be more efficient at meso- than at thermophilic temperatures—at least in batch reactors [21]. However, the preceding study focused on the degradation of aromatic amino acids (deriving from proteinaceous materials); a closer look on the degradation of lignin intermediates in terms of phenyl acid formation as well as microbial community dynamics is still missing.

In the present study, mesophilic as well as thermophilic microbial communities were fed with lignin intermediates under different overload conditions to (i) initiate anaerobic phenyl acid formation during the start-up phase of anaerobic lignin intermediate degradation, (ii) evaluate microbial community shifts during the formation/degradation of phenyl acids, and (iii) link the formation and/or degradation of PAA, PPA, and PBA to specific taxa, metabolic pathways, and enzymes.

## Results

### Mesophilic reactors

#### *Methane production*, *VFA concentrations*, *and pH*

**Fig. 1:**
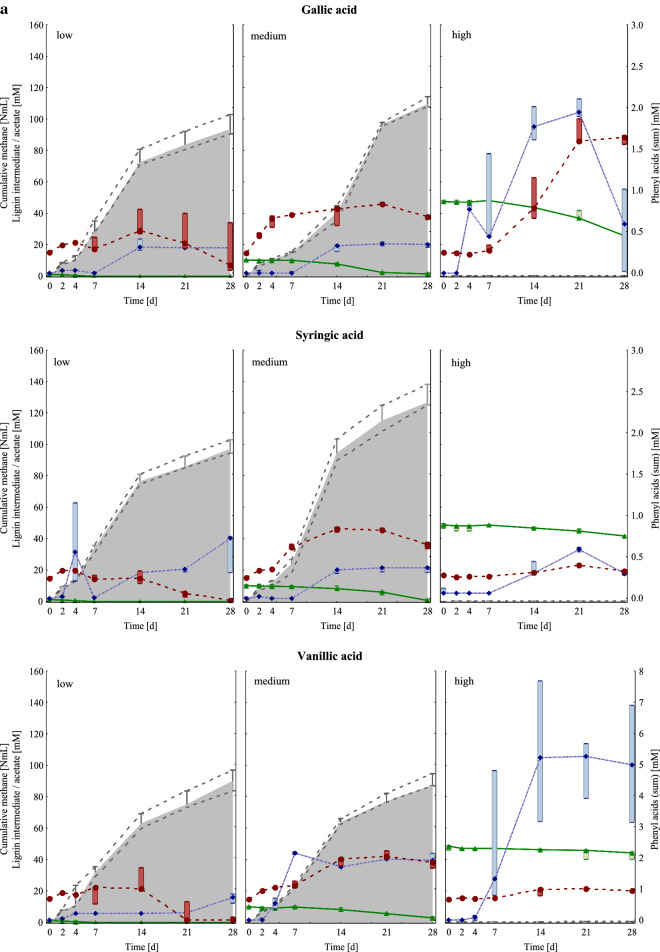

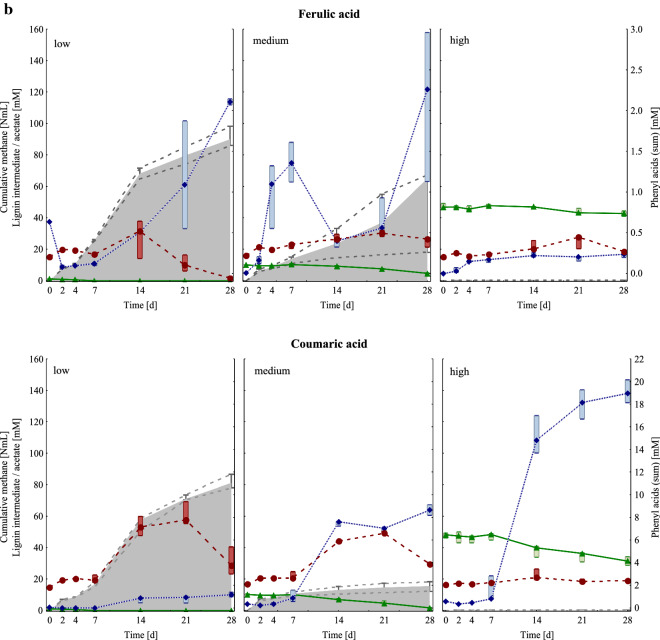
**a** Cumulative methane production (NmL) in grey, concentrations of lignin intermediates (mM) in green (triangle), sum of phenyl acids (mM) in blue (diamond), and acetate concentrations (mM) in red (circle) of mesophilic samples fed with gallic (upper row), syringic (middle row), and vanillic acid (lower row) under low (left column), medium (middle column), and high (right column) overload conditions from day 0 to day 28. Marker points and boxes show median and percentiles (25–75%), respectively. **b** Cumulative methane production (NmL) in grey, concentrations of lignin intermediates (mM) in green (triangle), sum of phenyl acids (mM) in blue (diamond), and acetate concentrations (mM) in red (circle) of mesophilic samples fed with ferulic (upper row) and coumaric acid (lower row) under low (left column), medium (middle column), and high (right column) overload conditions from day 0 to day 28. Marker points and boxes show median and percentiles (25–75%), respectively

Cumulative methane production and acetate concentrations are depicted in Fig. 1. For further results regarding volatile fatty acid (VFA) concentrations and pH values, please refer to Additional file 1: Table S1. With regard to the mesophilic controls, which produced up to 85 NmL methane (approx. 80% of the theoretical methane production) within 28 days, no significant differences in biogas production could be observed when the lignin intermediates were added under low carbon load (LCL) conditions. The highest cumulative (cum) methane production after 28 days could be observed in syringic acid samples under medium carbon load (MCL) conditions and in gallic acid MCL samples (130 ± 7.08 and 111 ± 3.40 NmL CH_4_ cum, respectively), followed by syringic acid LCL and gallic acid LCL samples (98.1 ± 4.38 and 95.6 ± 6.41 NmL CH_4_ cum, respectively). Within the MCL samples, significant differences in methane production could be observed for different lignin intermediates: Coumaric acid MCL samples produced significantly less methane (15.2 ± 3.10 NmL CH_4_) than syringic acid samples within 28 days. Hardly any methane was formed in samples under high carbon load (HCL) conditions, irrespective of the added lignin intermediate (Fig. 1).

A steep increase in acetate concentration could be observed in gallic acid HCL samples from day 7 onwards and reached its maximum on day 21 (90.2 ± 8.59 mM acetate, Fig. 1a). Coumaric LCL samples also showed quite high acetate concentrations on day 21 (60.7 ± 7.49 mM acetate, Fig. 1b). The acetate concentration in the control samples (20.2 ± 1.75 mM) peaked on day 14 and subsequently declined afterwards.

All variants started with a pH of 7.0. The pH slightly increased in the control samples up to 7.5 (day 28). Except for coumaric acid MCL samples, which showed a pH of 6.5 on day 28, all LCL and MCL samples exhibited a pH of 7.0 at the end of the incubation period; the lowest pH throughout the incubation period was 6.5 for LCL and 6.0 for MCL samples. In HCL samples, the pH decreased to 5.5 in gallic and ferulic acid samples, and to 5.0 in syringic, vanillic, and coumaric acid samples (Additional file 1: Table S1).

#### *Lignin intermediates and phenyl acid concentrations*

The concentrations of the respective lignin intermediates as well as methane production and phenyl acid sum concentrations are shown in Fig. 1. For a detailed depiction of each measured phenyl acid concentration, please refer to Additional file 1: Tables S2 and S3. Under LCL conditions, all lignin intermediates were degraded to smaller phenyl acids or non-aromatic molecules (Additional file 1: Tables S2 and S3, Fig. 1). Considerable differences in the degradation rate could be observed between the variants under MCL and HCL conditions. Under MCL conditions, 92% of the syringic acid was degraded to smaller molecules, followed by gallic acid (86%), coumaric acid (85%), vanillic acid (71%), and ferulic acid (54%). Under HCL conditions, a similar trend could be seen: the highest degradation rate was found in gallic acid samples (44%), followed by coumaric acid (34%), syringic acid (14%), vanillic (10%), and ferulic acid samples (10%).

The highest phenyl acid (sum) concentrations could be shown in coumaric HCL samples, especially from day 14 onwards. On day 28, a phenyl acid (sum) concentration of 19.1 ± 0.99 mM represented the climax (Fig. 1b). Coumaric acid samples under MCL conditions also showed an increase in phenyl acid (sum) concentration over time (8.66 ± 0.46 mM on day 28), followed by vanillic HCL samples (5.35 ± 2.25 mM on day 14). In control samples, the phenyl acid (sum) concentration ranged from 0.00 mM on day 0 to 0.33 ± 0.06 mM on day 14; these phenyl acids presumably derived from aromatic precursor remains, which were introduced with the inoculum.

#### *Microbial community composition*

After subsampling, 1050 operational taxonomic units (OTUs) remained for mesophilic samples. The removal of OTUs with a total read abundance below 50 resulted in a reduction to 343 OTUs. The most abundant phyla were *Bacteroidetes*, *Firmicutes*, *Chloroflexi*, and *Cloacimonetes*. The orders *Bacteroidales*, *Clostridiales*, *Anaerolineales*, *Cloacimonadales*, *Sphingobacteriales*, *Synergistales*, *Methanosarcinales*, *Betaproteobacteriales*, *Syntrophobacterales*, *Clostridia* DTU014, *Spirochaetales*, and *Caldatribacteriales* dominated in mesophilic samples. A detailed, interactive depiction of the microbial community composition of each variant on day 0, 14, and 28 can be looked up in Additional file 1: Figure S2).

**Fig. 2:**
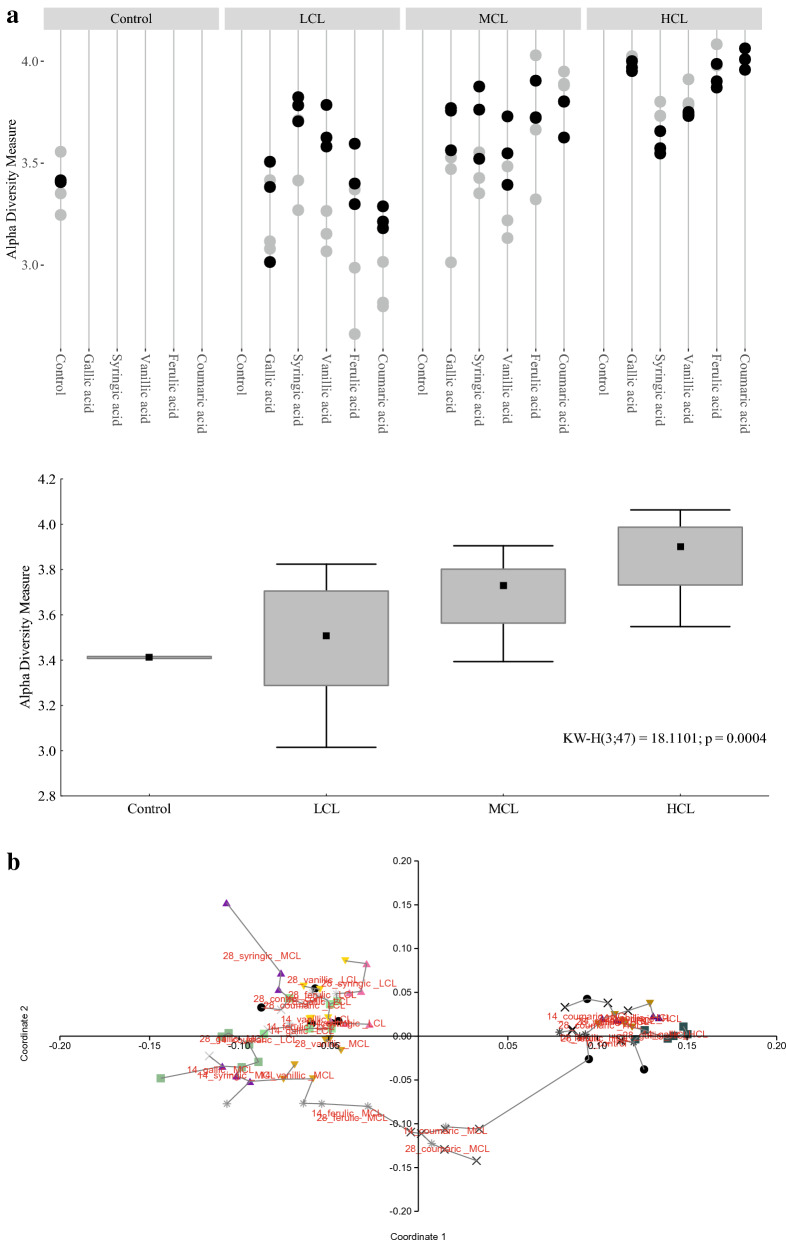
**a** Top: Shannon diversity index for mesophilic controls (1st column) and samples under LCL (2nd column), MCL (3rd column), and HCL (4th column) conditions. Results of day 14 and 28 are presented in grey and black, respectively. Bottom: Boxplots show diversity results of controls and LCL, MCL, and HCL samples of day 28. **b** NMDS analyses of mesophilic OTUs of the control as well as of the gallic-, syringic-, vanillic-, ferulic-, and coumaric acid samples at low (LCL), medium (MCL), and high (HCL) overload conditions of day 0, 14, and 28. The samples were connected to generate a minimal spanning tree. The *x*-axis is lengthened for better visualisation. The Shepard plot shows a stress value of 0.15

The alpha diversity of mesophilic samples was especially high at HCL conditions compared with the controls, LCL, and MCL samples of day 14 and 28 (Fig. 2a). As shown in the non-metric multidimensional scaling (NMDS) ordination (Fig. 2b), all HCL samples of day 14 and 28, as well as the controls of day 0 built a tight cluster, which was distinctly dissimilar from other samples/clusters. The next most similar objects to this cluster were coumaric acid MCL samples, followed by ferulic acid MCL samples of day 14 and 28, respectively. Moreover, control and LCL samples of day 14 and 28, as well as gallic and vanillic acid MCL samples of day 28 were closely ordinated (Fig. 2b) indicating similar community compositions.

**Fig. 3:**
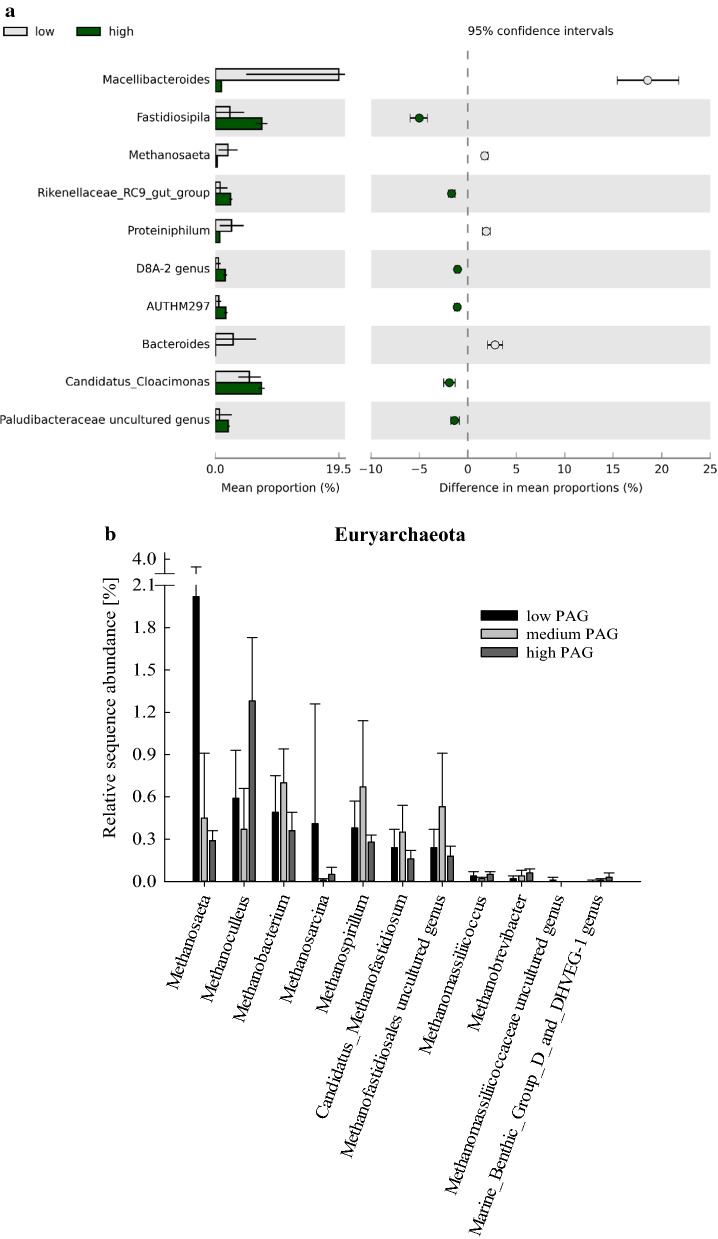
**a** Extended error bars showing mean sequence proportions (%) of mesophilic genera of the low (*n* = 82) and of the high (*n* = 6) PAG samples of day 0, 14, and 28. Due to the high diversity, only genera showing significant differences and an effect size > 1 are depicted. **b** Relative sequence abundances (%) of mesophilic methanogens of the low, medium, and high PAG. Bars and whiskers represent means and standard deviations, respectively

Regarding the microbial community composition, the phylum *Bacteroidetes* significantly decreased from the low (40.7 ± 12.0%), to the medium (26.3 ± 3.22%), to the high phenyl acid group (PAG) (26.2 ± 2.91%), whereas *Firmicutes* gradually increased (p < 0.05) from the low (16.4 ± 5.34%), to the medium (19.4 ± 2.56%), to the high PAG (24.7 ± 1.62%). The phylum *Euryarchaeota* also decreased with phenyl acid formation (4.44 ± 1.93%, 3.14 ± 1.12%, and 2.75 ± 0.63% in the low, medium, and high PAGs, respectively). On genus level, the mean relative abundance of *Anaerolineaceae* ADurb.Bin120 genus (7.51 ± 1.80%), *Fastidiosipila* spp. (7.32 ± 0.93%), and Candidatus *Cloacimonas* (7.29 ± 0.52%) were highest in the high PAG samples (*n* = 6), whereas *Macellibacteroides* spp. was the most abundant genus in low PAG samples (19.5 ± 14.6%), as shown in Fig. 3a. *Anaerolineaceae* ADurb.Bin120 was dominating in all lignin intermediate variations and was also a significant part of the core microbiome of high PAG samples (Table 2). *Fastidiosipila* spp. was also highly abundant at HCL conditions, especially in ferulic and coumaric acid samples (Additional file 1: Figure S1b). Moreover, *Fastidiosipila* spp. was a member of the core microbiome and a linear discriminant analysis effect size (*LEfSe*) biomarker for the high PAG (Table 2). The genus *Lactobacillus* was highly abundant in gallic and syringic acid HCL (and partly in vanillic acid HCL), but not in ferulic or coumaric acid HCL samples (Additional file 1: Figure S2).

In the control samples on day 0, which represent the native mesophilic community, *Methanosaeta* spp. was the most abundant methanogen (mean relative abundance: 1.5%), followed by two genera of the order *Methanofastidiosales* (0.8%), and *Methanospirillum* spp. (0.7%). Irrespective of the lignin intermediate, *Methanosaeta* spp. was the dominant methanogen in control and LCL samples of day 14 and 28. In MCL samples, a clear dominance of *Methanosaeta* spp. could not be observed. For instance, in gallic and syringic acid MCL samples, a mean relative abundance of 2% could be shown for *Methanosarcina* spp. By contrast, *Methanoculleus* spp. was the dominating methanogen in gallic acid and coumaric acid HCL samples on day 14 and 28 (1%) and was also a significant *LEfSe* biomarker when high phenyl acid concentrations were observed (Table 2). The relative abundances of methanogens of the respective PAGs are depicted in Fig. 3b.

### Thermophilic communities

#### *Methane production*, *VFA concentrations*, *and pH*

**Fig. 4:**
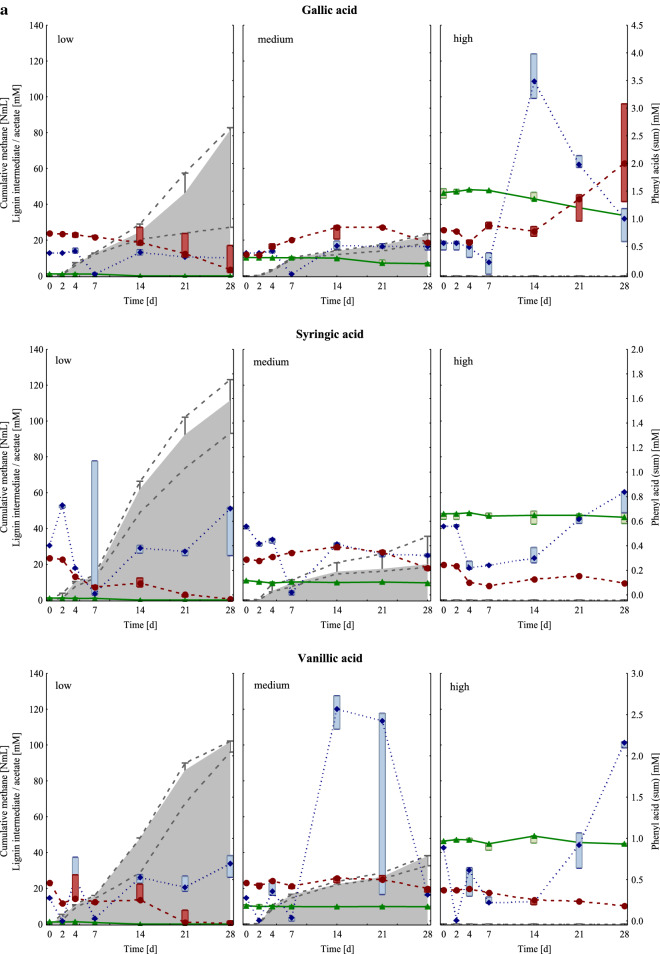

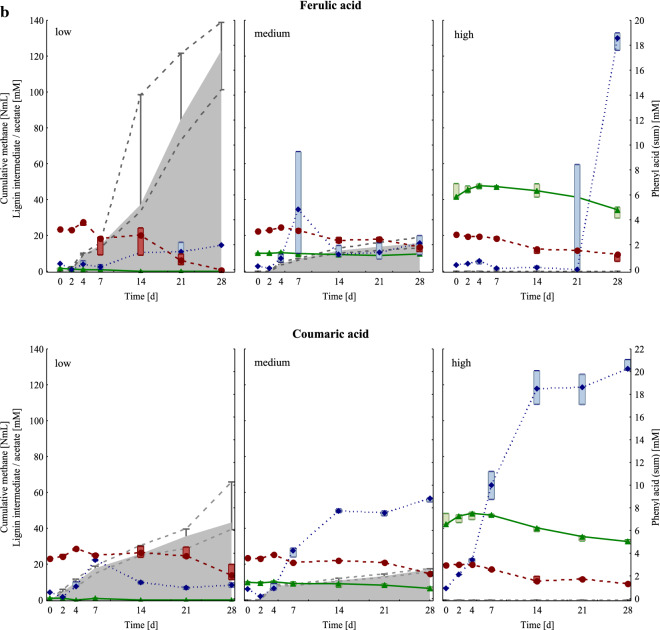
**a** Cumulative methane production (NmL) in grey, the concentrations of lignin intermediates (mM) in green (triangle), phenyl acids (mM) in blue (diamond), and acetate (mM) in red (circle) of thermophilic samples fed with gallic (upper row), syringic (middle row), and vanillic acid (lower row) under low (left column), medium (middle column), and high (right column) overload conditions from day 0 to 28. Marker points and boxes show median and percentiles (25–75%), respectively. **b** Cumulative methane production (NmL in grey), the concentrations of lignin intermediates (mM) in green (triangle), phenyl acids (mM) in blue (diamond), and acetate (mM) in red (circle) of thermophilic samples fed with ferulic (upper row) and coumaric acid (lower row) under low (left column), medium (middle column), and high (right column) overload conditions from day 0 to 28. Marker points and boxes show median and percentiles (25–75%), respectively

Cumulative methane production and acetate concentrations of thermophilic samples are depicted in Fig. 4. For further results regarding VFA concentrations and pH values, please refer to Additional file 1: Table S4. Generally, the cumulative methane production was significantly lower in HCL than at all other variants. Moreover, the difference in cumulative methane production was also significant between LCL than MCL samples. The highest cumulative methane production could be observed in ferulic acid LCL (121 ± 18.8 NmL CH_4_ cum), syringic acid LCL (109 ± 15.1 NmL CH_4_ cum), and in vanillic acid LCL samples (100 ± 3.41 NmL CH_4_ cum). The control showed a cumulative methane production of 95.9 ± 19.9 NmL (approx. 86% of the theoretical methane production). All other variants showed a lower methane production than the control. No methane was produced in HCL samples, irrespective of the substrate used.

Acetate accumulated in gallic HCL samples and reached a final concentration of 66.8 ± 27.5 mM. Generally, a relevant accumulation of acetate could only be detected in gallic HCL samples, whereas the acetate concentrations stagnated or decreased in all other variants over time (Fig. 4).

All variants started with a pH of 7.0. Similar to mesophilic control samples, the pH slightly increased to 7.5 in thermophilic control samples from day 2 onwards. In LCL samples, the pH remained at 7.0 (except for gallic LCL samples on day 4: pH 6.5). In MCL samples, the pH decreased to 6.5 in syringic and ferulic acid samples and to 6.0 in gallic acid samples. The pH in vanillic and coumaric acid MCL samples remained at 7.0, whereas a pH of 5.0 was observed in all HCL samples at the end of the incubation period (Additional file 1: Table S4).

#### *Lignin intermediates and phenyl acid concentrations*

The concentrations of lignin intermediates as well as phenyl acid sum concentrations are shown in Fig. 4. For a detailed depiction of each measured phenyl acid concentration, please refer to Additional file 1: Tables S5 and S6. Under LCL conditions, lignin intermediates were degraded to smaller phenyl acids or non-aromatic compounds (Fig. 4, Additional file 1: Tables S5 and S6). Significant differences could be observed regarding the degradation rates of MCL and HCL variants: Under MCL conditions, 35% of gallic and coumaric acid content was degraded, followed by 11% of the syringic acid, 6% of the vanillic acid, and 1% of the ferulic acid content. Under HCL samples, 28% of the gallic acid, 25% of the coumaric acid, 24% of the ferulic acid, 4% of the vanillic acid, and 2% of the syringic acid content was degraded within 28 days of incubation (Fig. 4).

Phenyl acids (sum) accumulated in coumaric HCL samples from day 0 onwards and reached a concentration of 20.5 ± 0.50 mM on day 28 (Fig. 4b). The second highest phenyl acid (sum) concentration at the end of the incubation period could be observed in ferulic acid HCL samples (18.4 ± 0.70 mM), followed by coumaric MCL samples (8.74 ± 0.18 mM), as shown in Fig. 4b. In the control samples, phenyl acids reached a maximum sum concentration of 0.42 mM on day 4.

#### *Microbial community composition*

A total of 856 OTUs remained for thermophilic samples after subsampling. The removal of OTUs with a total read abundance below 50 resulted in a reduction to 195 OTUs. Over all thermophilic samples, the most abundant phyla were *Firmicutes*, *Bacteroidetes*, *Thermotogae*, *Euryarchaeota*, *Atribacteria*, and *Tenericutes*. On order level, *Clostridiales*, *Bacteroidales*, *Thermoanaerobacterales*, *Petrotogales*, *Sphingobacteriales*, *DTU014* (*Clostridia*), *Bacillales*, *MBA03* (*Clostridia*), *Methanomicrobiales*, *Caldatribacteriales*, an uncultured order of *Firmicutes*, and *Izimaplasmatales* were most dominant. A detailed, interactive depiction of the microbial community composition of each thermophilic variant on day 0, 14, and 28 can be looked up in Additional file 1: Figure S5.

**Fig. 5:**
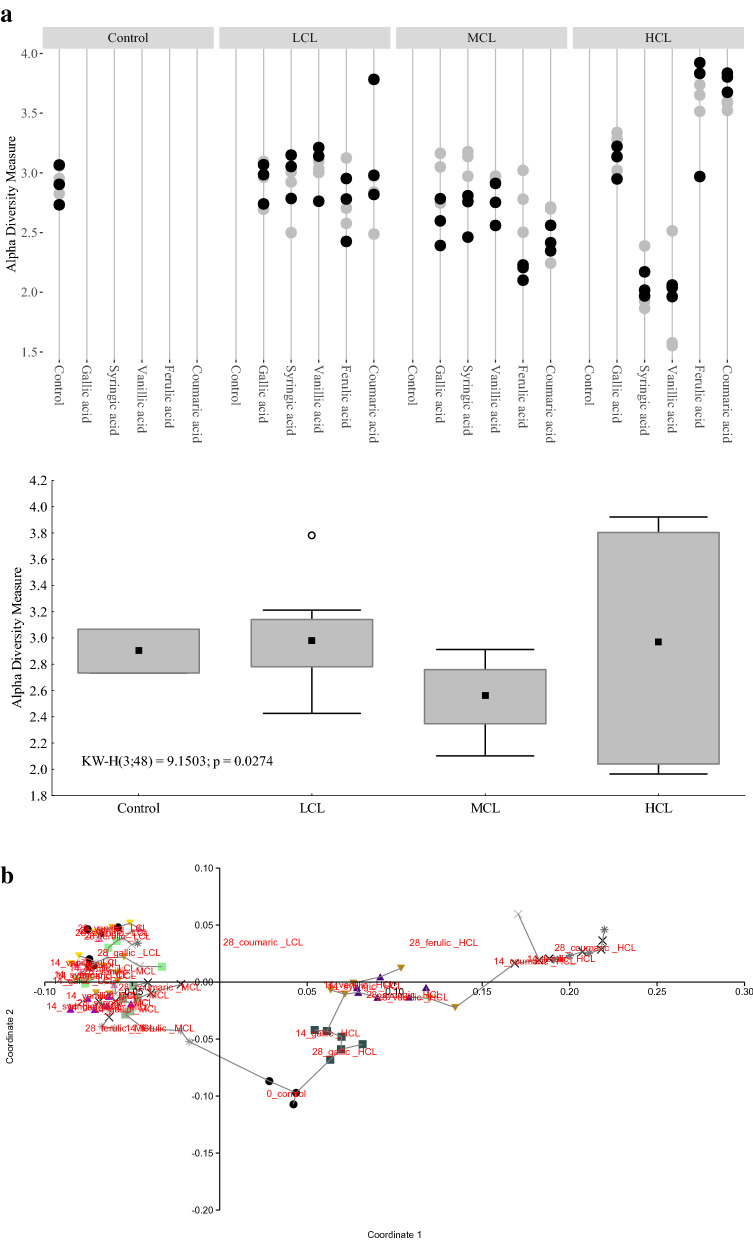
**a** Top: Shannon diversity index for thermophilic controls (1st column) as well as samples under LCL (2nd column), MCL (3rd column), and HCL (4th column) conditions. Results of day 14 and day 28 are presented in grey and black, respectively. Bottom: Boxplots show diversity results of controls and LCL, MCL, and HCL samples of day 28. **b** NMDS analyses of thermophilic OTUs of the control as well as of the gallic-, syringic-, vanillic-, ferulic-, and coumaric acid samples at low (LCL), medium (MCL), and high (HCL) overload conditions of day 0 (control), 14, and 28. The samples are connected to generate a minimal spanning tree. The *x*-axis is lengthened for better visualisation. The Shepard plot shows a stress value of 0.12

The alpha diversity was relatively low in syringic and vanillic HCL samples, whereas the microbial diversity was exceptionally high in ferulic and coumaric HCL samples (Fig. 5a). As shown in Fig. 5b, LCL and MCL samples of day 14 and 28 build a tight cluster, which is distinctly dissimilar to other samples or clusters. The next most similar variant to this cluster is the control on day 0. HCL samples can be divided into 3 sub-clusters, whereby ferulic and coumaric acid HCL samples, syringic and vanillic acid HCL samples, and gallic acid HCL samples build respective sub-clusters. Gallic acid samples are the next most similar variants to the controls on day 0 (Fig. 5b).

**Fig. 6:**
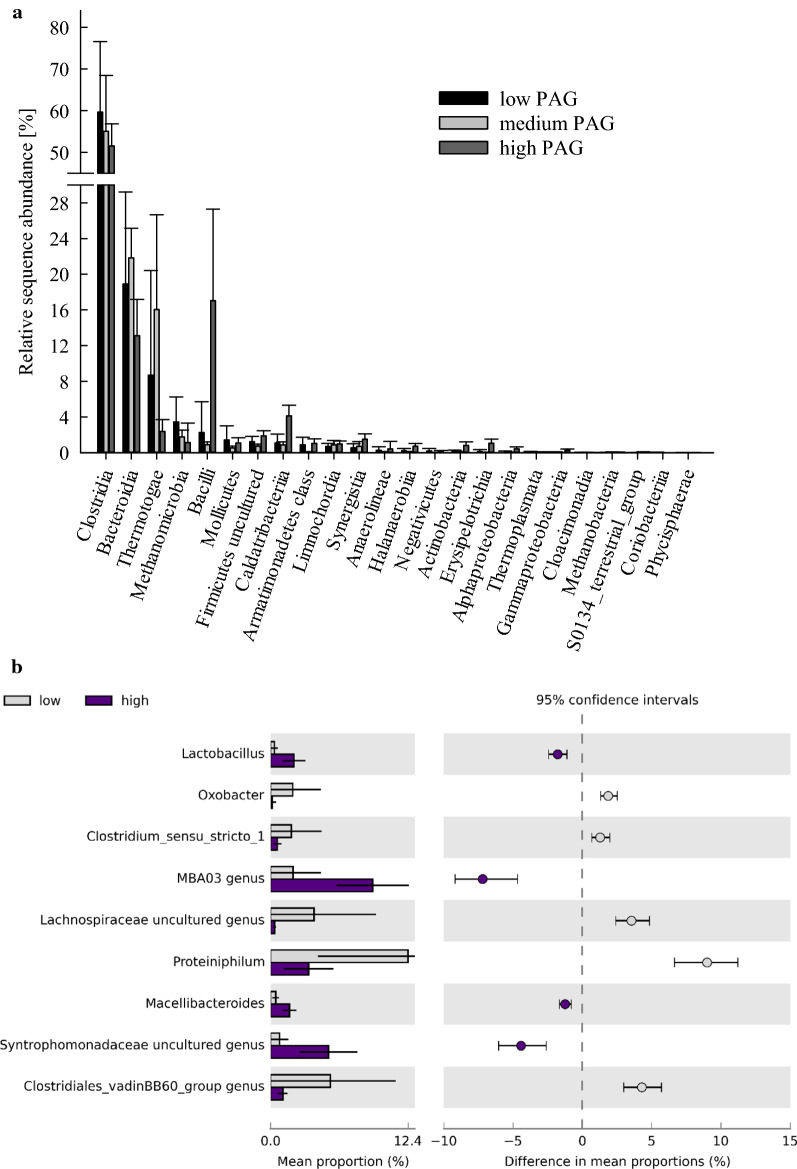
**a** Relative sequence proportions (%) of all thermophilic classes of low (black), medium (light grey), and high (dark grey) PAG samples. Bars and whiskers show means and standard deviations, respectively. **b** Extended error bars showing sequence proportions (%) of thermophilic genera of the low (*n* = 84) and of the high (*n* = 9) PAG samples of day 0, 14, and 28. Due to the high diversity, only genera showing significant differences and an effect size > 1 are depicted

When specifically looking on phenyl acid formation, the relative abundance of the phyla *Atribacteria*, *Armatimonadetes, Gemmatimonadetes*, *Synergistetes*, *Halanaerobiaeota*, *Actinobacteria*, and *Proteobacteria* was significantly higher in the high PAG than in the other PAGs; however, their abundance was relatively low compared with phyla like *Firmicutes* (low: 64.1 ± 17.9%, medium: 57.7% ± 13.3%, and high: 72.7 ± 7.80%) and *Bacteroidetes* (low: 18.9 ± 10.3%, medium: 21.8 ± 3.34%, and high: 13.1 ± 4.05%). The relative abundance of the class *Bacilli* was significantly higher in high PAG samples and reached a relative abundance of 17.0 ± 10.3% (Fig. 6a) and is thus the second highest class in high PAG samples after *Clostridia* (51.5 ± 5.28%).

The genera *Proteiniphilum* (12.5 ± 8.15%) and *Caldicoprobacter* (10.6 ± 11.3%) were the most abundant genera in low PAG samples, whereas *Syntrophaceticus* spp. and MBA03 genus (*Clostridia*) were the most abundant genera over all high PAG samples (10.0 ± 5.29% and 9.25 ± 3.29%, respectively). However, *Proteiniphilum* spp. was still a member of the core microbiome of high PAG samples (Table 3). Compared with low PAG samples, the genera *Macellibacteroides*, *Lactobacillus*, MBA03 genus (*Clostridia*), and an uncultured genus of family *Syntrophomonadaceae* were significantly increased in the high PAG samples (Fig. 6b). The latter three were also significant *LEfSe* biomarker for the high PAG (Table 3). When looking on the respective intermediates, *Syntrophaceticus* spp. was also the most abundant genus in coumaric HCL samples (13.7 ± 0.91%), whereas *Thermoanaerobacterium* spp. was by far the most dominant genus in vanillic and syringic acid HCL samples (63.4 ± 7.90% and 60.4 ± 4.25%, respectively), as shown in Additional file 1: Figures S5a and b. In gallic and ferulic HCL samples, an uncultured genus of the family *Lachnospiraceae* and MBA03 genus (*Clostridia*) were the most abundant microorganisms (20.5 ± 5.98% and 10.4 ± 4.50%, respectively).

**Fig. 7:**
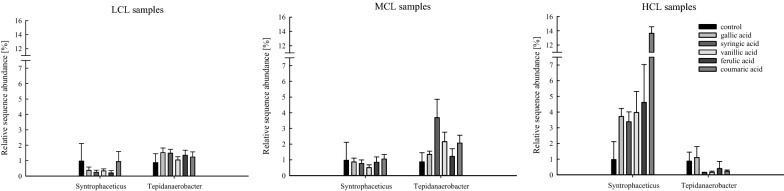
Relative sequence abundance of the two syntrophic acetate oxidising bacteria (SAOB) *Syntrophaceticus* spp. and *Tepidanaerobacter* spp. in thermophilic controls as well as in thermophilic LCL (left), MCL (middle), and HCL (right) samples. Bars and whiskers represent mean and standard deviation, respectively

*Syntrophaceticus* spp. and *Tepidanaerobacter* spp., known syntrophic acetate oxidising bacteria (SAOBs), were quite abundant in thermophilic samples (Fig. 7). Especially in HCL samples, *Syntrophaceticus* spp. was an important member of the respective microbiome and reached a mean relative abundance ranging from 3 to 14%, while the relative abundance of *Tepidanaerobacter* spp. ranged from 0.1 to 1.1% (Fig. 7).

## Discussion

### Mesophilic communities

In general, mesophilic samples showed a higher diversity than thermophilic samples, as shown in Figs 2a and 5a and in a summarised form in Additional file 1: Figure S7. This trend was also observed in previous studies [19, 22]. Functional and microbial redundancies, as often observed in microbiologically diverse environments, can lead to relatively high process robustness, but could also indicate a stressed microbial community [23]. The higher microbial diversity in mesophilic samples might have been beneficial for degrading lignin intermediates to methane, especially at MCL conditions (Figs. 1 and 4).

Under LCL conditions, all lignin intermediates were degraded (Fig. 1); however, it can be assumed that degradation products of the lignin intermediates were partly stuck in upstream degradation steps. *Methanosaeta* spp. was the dominating methanogen in mesophilic control and LCL samples, whereby the diversity of methanogens was higher in the native sludge (control on day 0, Additional file 1: Figure S2). *Methanosaeta* spp. is an acetoclastic methanogen with a high affinity towards acetate (< 1 mM) [4, 24], thus a typical methanogen in (mesophilic) LCL systems like wastewater treatment plants [25]. When acetate levels are high (> 1 mM) and the ammonium concentrations are low, *Methanosarcina* spp., another acetoclastic methanogen, has an advantage over *Methanosaeta* due to its high growth rates [4] and its *heavy-duty* character [19, 26]. In the present study, most strikingly, *Methanosaeta* spp. was able to dominate in mesophilic control and LCL samples, even though acetate concentrations were relatively high from the beginning (about 20 mM). Studies reporting competitiveness of *Methanosaeta* spp. at higher acetate levels are rare [27, 28]; therefore, the results of this study indicate that acetate levels may not be necessarily decisive and that other factors like aromatic compound concentrations might be more crucial for which acetoclastic genus is prevailing.

At MCL conditions, methane formation was still or even better functioning than at LCL conditions; moreover, each lignin intermediate had a different impact on the overall methane production. At MCL conditions, an input of gallic and syringic acid was shown to increase biogas production in mesophilic samples (Fig. 1a), whereas the methane production was successively lower when ferulic and coumaric acid were added (Fig. 1b). The toxicity of aromatic compounds (for acetoclastic methanogens) was shown to increase with the hydrophobicity of the compound [29] and length of the aliphatic side chain [30]. Ferulic and coumaric acid have a longer side chain than the other lignin intermediates (Table 1); therefore, the restricted methane production in ferulic, and especially in coumaric acid MCL samples is plausible. Moreover, polar compounds like carboxyl- and hydroxyl-groups can counteract the overall toxicity [29, 30]. Gallic acid has three hydroxyl- and one carboxyl-group (Table 1), and represents the highest oxidised lignin intermediate of the present study (based on *Buswell-Boyle* calculations [31], the anaerobic degradation of gallic acid theoretically results in a molar CH_4_:CO_2_ ratio of 0.75, please also refer to its structure in Table 1). The AD of gallic acid at MCL conditions led to the second highest methane formation in mesophilic samples. Syringic acid includes two methoxy-, one hydroxyl-, and one carboxyl-group (Table 1), and led to the highest methane formation in mesophilic MCL samples after 28 days. In previous studies, methoxy-groups were considered to increase the toxicity of an aromatic compound towards acetoclastic methanogens [30]. In the present study, however, the acetoclastic genus *Methanosaeta* was the most abundant methanogen in mesophilic MCL samples when at least one methoxy-group was attached to the lignin intermediate. The assumption that methoxy-groups can be beneficial for (mesophilic) acetoclastic methanogens is in accordance with previous studies, which concluded that O-demethylation is primarily done by acetogenic (thus acetate providing) microorganisms [20, 32]. Acetogens are a phylogenetically incoherent and highly diverse group though [33], which makes it difficult to associate acetogenesis with specific mesophilic genera. In gallic and coumaric acid MCL samples, which contained no methoxy-groups, *Methanosaeta* spp. was replaced by *Methanosarcina* spp. and hydrogenotrophic methanogens, respectively (Additional file 1: Figure S2b). Not only methanogenesis but also acetogenesis was functioning in all MCL conditions as (i) acetate was provided to acetoclastic methanogens (Additional file 1: Figure S2) and (ii) acetate accumulated in coumaric acid samples (Fig. 1); however, first impairments were probably the case as butyrate and i-butyrate accumulated (Additional file 1: Table S1). The effect of pH in MCL samples is considered marginally as the buffer capacity of the medium was sufficient to keep the pH mostly at 7.0. Even when the pH temporarily dropped to slightly acidic conditions (pH 6.0–6.5, Additional file 1: Table S1), a pure effect of pH due to the lignin intermediate addition is unlikely: While methane formation was indeed restricted in ferulic and coumaric acid MCL samples (Fig. 1), gallic, syringic, and vanillic acid MCL even produced more methane than the control (thus > 85 NmL CH_4_).

**Table 1:** Overview of the intermediate variants used for the present study

Lignin intermediates	Structure	Lignin intermediate		Overload group
		mmol reactor^−1^	mM	
Control	–	0	0	Control
Gallic acid		0.34	1	Low
		3.36	10	Medium
		16.8	50	High
Syringic acid		0.43	1	Low
		4.32	10	Medium
		21.6	50	High
Vanillic acid		0.38	1	Low
		3.84	10	Medium
		19.2	50	High
Ferulic acid		0.48	1	Low
		4.80	10	Medium
		24.0	50	High
Coumaric acid		0.43	1	Low
		4.32	10	Medium
		21.6	50	High

The chemical structures were assessed via *ChemDraw*® JS. The lignin intermediate concentrations were chosen based on previous investigations [56, 77]

HCL samples in general showed a relatively high microbial diversity (Fig. 2a), which was comparable with the microbial diversity in control samples on day 0 (Fig. 2b). This indicates that specific microbial groups could not be dominating in HCL samples due to the unfavourable conditions; therefore, the mesophilic microbial composition and abundance did not considerably change in HCL samples over time. High lignin intermediate additions indeed inhibited methane generation (Fig. 1) and participating methanogens (Additional file 1: Figure S2) due to multifactorial effects caused by the pH drop (pH 5.0–5.5, Additional file 1: Table S1) and the structural characteristics of the respective lignin intermediate (Table 1). Within the HCL samples, lignin intermediates had different effects on the degradation phases prior to methanogenesis: In gallic acid HCL samples, gallic acid and phenyl acid concentrations decreased (at the end of the incubation period), whereas acetate accumulated up to a concentration of approx. 90 mM (Fig. 1a). Apparently, inhibitory effects could only be observed at the methanogenesis stage, while preceding stages were less impacted. In all other HCL samples, acetate concentrations remained low (Fig. 1). As acetoclastic methanogens and acetate oxidising bacteria were clearly inhibited and i-butyrate/butyrate accumulated (Additional file 1: Table S1, Figure S2), it can be assumed that not only methanogenesis but also acetogenic processes were malfunctioning in syringic, vanillic, ferulic, and coumaric acid HCL samples. The slightly better conditions in gallic acid HCL samples are further supported by the fact that the orthology counts for the fatty acid metabolism pathway (ko01212), carbon fixation pathways (ko00720), and aromatic compound turnover pathways (ko00940, ko01220, and ko00362) were higher in gallic than in other HCL samples (Additional file 1: Figure S3). Phenyl acids did not accumulate in syringic (two methoxy-groups) and ferulic acid (one methoxy-group) HCL samples, while phenyl acids (mainly PAA) accumulated in coumaric and partly in gallic acid HCL samples (no methoxy-groups, Fig. 1, Additional file 1: Tables S2, S3). In vanillic acid HCL samples (one methoxy-group), phenyl acids also accumulated but contained mainly PPA and PBA. These are interesting dynamics, which show that the functional groups of lignin intermediates also have an impact on higher degradation phases; this has to be elucidated in depth in future studies.

An accumulation of mono-aromatic compounds with quite long aliphatic side chains but otherwise no attached functional groups (like PAA, PPA, and PBA) might indicate that (i) the respective microbial community is capable of cata- and/or anabolically use the lignin intermediates, (ii) there is yet a metabolic bottle-neck as the phenyl acids are not (effectively) processed [30]. Even though this still has to be validated, the results (of the meso- and the thermophilic samples) support the approach to monitor PAA, PPA, and PBA concentrations to ensure process stability during biogas formation, especially in the start-up phase [12, 13, 17, 19]. Many microorganisms (biomarkers) for high phenyl acid formation were fermentative bacteria and/or involved in acidification and VFA production (Table 2 and Fig. 3a); this indicates that phenyl acids were formed during the acido- and acetogenesis phase. *Fastidiosipila* spp., for instance, was both a *LEfSe* biomarker and a core member of the mesophilic high PAG microbiome (Table 2 and Fig. 3a). In previous investigations, this genus was associated with butyrate and acetate formation during mesophilic AD [34]. Moreover, *Fastidiosipila* spp. positively correlated with concentrations of phenols, indoles, and various VFAs in studies investigating pig slurry [35]. Two species of the genus *Syntrophomonas* (Table 2) were shown to degrade butyrate in syntrophic associating with hydrogenotrophic methanogens; however, the organisms were not able to degrade branched fatty acids or benzoate [36, 37].

**Table 2:** Mesophilic core microbiome and *LEfSe* biomarker for the high PAG samples (*n* = 6)

Core microbiome	*LEfSe* biomarker (LDA score ≥ 4)
*Anaerolineaceae* uncultured genus *Lentimicrobiaceae* genus *Bacteroidetes* vadinHA17 genus ***Clostridia DTU014 genus*** *Candidatus Cloacimonas* *Anaerolineaceae* ADurb.Bin120 genus *Cloacimonadaceae* W5 genus *Prolixibacteraceae* uncultured genus ***Fastidiosipila*** *Sedimentibacter* ***Syntrophomonas*** *Rikenellaceae* Blvii28 wastewater-sludge group *Rikenellaceae* RC9 gut group *Petrotogaceae* AUTHM297 genus *Spirochaetaceae* uncultured genus *Paludibacteraceae* uncultured genus *Clostridia* D8A-2 genus	***Fastidiosipila*** Candidatus *Cloacimonas* ***Clostridia DTU014 genus*** *Lentimicrobiaceae* genus *Rikenellaceae* Blvii28 wastewater-sludge group *Rikenellaceae* RC9 gut group *Clostridia* D8A-2 genus *Paludibacteraceae* uncultured genus *Petrotogaceae* AUTHM297 genus ***Syntrophomonas*** ***Kiritimatiellae WCHB1-41 genus*** *Erysipelotrichaceae* UCG-004 genus *Pedosphaeraceae* genus *Bacteroidales* UCG-001 genus *Verrucomicrobiae* LD1-PB3 genus ***Methanoculleus*** *Gracilibacter* *Bacteroidales* M2PB4-65 termite group ***Dysgonomonadaceae uncultured genus*** *Spirochaetaceae* uncultured genus *Absconditabacteriales* (SR1) genus

Only *LEfSe* biomarkers with a linear discriminant analysis (LDA) score of 4 or higher are depicted. Genera in bold were previously associated with phenyl acid formation in a study using protein-rich substrates [21]

### Thermophilic communities

Surprisingly, the thermophilic microbial communities, which are adapted to HCL conditions [19, 38], could cope less with multifactorial effects caused by the respective lignin intermediates than the mesophilic microbial communities, which derived from an LCL system (Figs. 1, 4, Additional file 1: Figures S2, S5). The microbial diversity was generally lower in thermo- than in mesophilic communities (Additional file 1: Figure S7). This could indicate that the thermophilic community was very specialised, with a low microbial redundancy. The microbial communities were quite similar in LCL and MCL samples (Fig. 5b), whereas HCL conditions led to specific changes in the composition of microorganisms engaged in different degradation phases. Moreover, the microbial community compositions did also change with the respective lignin intermediate within the HCL group, as shown in Fig. 5b. This is further supported by the alpha diversity measurements at HCL conditions, which showed that the microbial diversity in gallic, ferulic, and coumaric acid fed reactors was high compared with the microbial diversity in reactors fed with syringic or vanillic acid (Fig. 5a)—a trend that was (although to a lower extent) also observed in mesophilic HCL samples (Fig. 2a).

Acetogenesis was functioning in thermophilic MCL samples, but showed first impairments as i-butyrate and butyrate accumulated (Fig. 4, Additional file 1: Table S4). Propionate accumulated (Additional file 1: Table S4) when phenyl acid concentrations increased in the first days of incubation, but were also effectively degraded a few days later, as shown in gallic acid HCL, vanillic acid MCL, and (to a lesser extent) in ferulic acid MCL samples (Fig. 4, Additional file 1: Table S4). This association (though less distinct) was also observed with mesophilic ferulic acid MCL samples (Fig. 1, Additional file 1: Table S1). Especially, gallic acid HCL samples showed a steep increase in propionate concentration from day 21 (6.32 ± 1.69 mM) to day 28 (34.2 ± 2.01 mM). These results indicated that propionate is formed during phenyl acid degradation; further investigations are needed though to verify the results and to elucidate exact degradation pathways. Methane formation in thermophilic MCL samples was significantly lower than in mesophilic equivalents (Figs. 1 and 4). The slightly acidic conditions in gallic, syringic, and ferulic MCL samples (Additional file 1: Table S4) could in part be responsible for the low methane formation in those samples; however, in vanillic and coumaric acid MCL samples, neutral conditions were prevalent, but the methane formation was restricted, as well. This shows that the low methane production in MCL samples was mainly caused by the respective lignin intermediate itself and less by secondary (pH) effects. While *Methanosarcina* spp. was the dominant acetoclastic methanogen in thermophilic control and LCL samples (Additional file 1: Figure S5b), syntrophic acetate oxidation (SAO)-induced hydrogenotrophic methanogenesis was common in thermophilic MCL samples (except for ferulic MCL on day 28, Fig. 7 and Additional file 1: Figure S5b). This is in accordance with (i) the high relative abundances of acetate producers like *Defluviitoga* spp. [39] or *Caldicoprobacter* spp. [40] (Additional file 1: Figure S5), thus with the assumption that acetogenesis was still functioning in MCL samples and (ii) previous studies, which concluded that SAO is energetically more favourable at thermo- than at mesophilic temperatures [41]. Acetoclastic methanogens in thermophilic systems seem to be exceptionally susceptible towards phenyl acids; however, *Methanosarcina* spp. was previously shown to have an essential role in stabilising reactors at overload conditions when ammonium concentrations were low [19, 26, 42]. The susceptibility of acetoclastic methanogens towards ammonium or aromatic compound might depend on multiple microbial, biochemical (e.g., pH, CO_2_, H_2_ partial pressure, and acetate concentrations), and thermo-dynamical properties [41, 43, 44], and is thus controversially discussed [4, 13, 17, 19]. The results of this study further indicate that SAOBs are less impacted by aromatic compounds and thus can step in and oxidise the acetate for the respective hydrogenotrophic partner. *Syntrophaceticus* spp. and *Tepidanaerobacter* spp. are two typical syntrophic acetate oxidising bacteria in methanogenic systems [4, 10]. In previous studies, the type of (proteinaceous) substrate determined which of these two SAOBs was dominating [21]. In the present study, the extent of the overload of the lignin intermediates had a strong influence (Fig. 7): In control and LCL samples on day 28, *Tepidanaerobacter* spp. was the dominant SAOB. Under MCL conditions, both SAOBs were competitive and also highly relevant for methane formation. Astonishingly, *Syntrophaceticus* spp. was by far the most dominant SAOB in HCL (and especially in coumaric acid HCL) samples, even though acidic conditions were prevalent (Additional file 1: Table S4) and methane production was thus inhibited (Fig. 4 and Additional file 1: Figure S5b). These results show interesting dynamics within the known SAOB member community; however, it is not clear to what extent parameters like aromatic compound, pH, ammonium [4, 10], and acetate concentration, carbon overload, or syntrophic partners have an influence on the prevalence of the respective SAOB. *Syntrophaceticus schinkii*, the only described species of this genus, is a mesophilic microorganism with a pH growth range from 6.0 to 8.0 [45]. The prevalence of *Syntrophaceticus* spp. in thermophilic samples under acidic conditions indicates that this microorganism could be another, yet unknown species of the very same genus. *Syntrophaceticus* spp. most probably grew also on other substrates like lactate or ethanol [45]. This is supported by the fact that i) the use of other electron acceptors like nitrate or sulphate during SAO is rather unlikely [45] as the orthology counts for the nitrogen and sulphur metabolism were low throughout the present study (Additional file 1: Figure S6) and ii) the abundance of the class *Bacilli* in general and of the order *Lactobacillales* (*Lactobacillus* spp. and *Jeotgalibaca* spp.) in particular was relatively high in ferulic and coumaric HCL samples as well as in high PAG samples (Fig. 6, Additional file 1: Figures S4b, and S5b).

The relative abundance of *Thermoanaerobacterium* spp. was astonishingly high (about 60%) in syringic and vanillic acid HCL samples on day 14 as well as on day 28. The next abundant genera (and also members of the class Clostridia) were *Syntrophaceticus* (45%), DTU014 (4%), and MBA03 (3%), as shown in Additional file 1: Figures S4a and S5. H_2_ concentrations in the headspace were also high (6–9%) in syringic and vanillic HCL samples from day 2 onwards, which might have been due to the capability of *Thermoanaerobacterium* spp. to produce H_2_ from various substrates even at low pH levels [46]. Moreover, the Kyoto Encyclopedia of Genes and Genomes (KEGG) enzyme analyses indicate that *Thermoanaerobacterium* spp. is also able to consume acetate via the intermediate glycin, which is used as electron donor or acceptor. This quite recently discovered strategy is used by some bacteria to utilise acetate while circumventing the carbonyl- and methyl cleavage of the conventional Wood–Ljungdahl pathway [47]. The orthology counts for the glycin dehydrogenase (subunit 1: K00282 and subunit 2: K00283) were indeed high in syringic and vanillic HCL samples (Additional file 1: Figure S8c), and could be mainly associated with *Thermoanaerobacterium thermosaccharolyticum*. Apparently, syringic and vanillic acid led to non-methanogenic, bacterial communities, which were unique but highly restricted and only capable of consuming easier degradable carbohydrates and VFAs with acetate as central intermediate (Figs. 4, 5, Additional file 1: Figures. S5, S8, Table S4). Phenyl acid concentrations slightly increased in syringic and vanillic HCL samples at the end of the incubation (Fig. 4) though. A direct involvement of *Thermoanaerobacterium* spp. in aromatic compound dynamics could not be proven; however, the restriction to syringic and vanillic acid HCL samples is still noteworthy.

The genera *Lactobacillus*, *Thermoactinomyces*, *Geobacillus*, *Ureibacillus*, and *Bacillus* spp., all members of the class *Bacilli*, were significant biomarkers for high phenyl acid formation (Table 3). *Thermoactinomyces mirandus* is the first (and so far only) described anaerobic member of the genus [48]. *Lactobacillus* spp. was associated with phenyl acid formation before [21]; moreover, lactic acid bacteria are known to produce PAA and phenyllactate [49, 50]. Degradation of aromatic compounds by *Bacillus* spp. was previously reported under aerobic conditions [51, 52]; a turnover of phenyl acids by *Bacillus* spp. is also plausible under anaerobic conditions as discussed before [19]. An association of the genera *Ureibacillus* and *Geobacillus* with phenyl acids dynamics (biomarkers, Table 3) could also be shown previously [19, 21].

**Table 3:** Thermophilic core microbiome and *LEfSe* biomarker for the high PAG (*n* = 9)

Core microbiome	*LEfSe* biomarker (LDA score ≥ 4)	
*Proteiniphilum* *Caldicoprobacter* *Lentimicrobiaceae* genus *Defluviitoga* ***Clostridia DTU014 genus*** *Hydrogenispora* *Candidatus Caldatribacterium* *Clostridia* MBA03 genus *Firmicutes* uncultured genus	*Lentimicrobiaceae* genus MBA03 genus (*Clostridia*) ***Bacillus*** ***Syntrophaceticus*** *Syntrophomonadaceae* uncultured genus ***Clostridia DTU014 genus*** Candidatus *Caldatribacterium* ***Geobacillus*** *Planifilum* ***Tissierella***	*Thermoactinomyces* *Sporosarcina* D8A-2 genus (*Clostridia*) ***Ureibacillus*** *Erysipelothrix* *Firmicutes* uncultured genus *Jeotgalibaca* ***Acetomicrobium*** ***Lactobacillus*** *Armatimonadetes* genus

Only *LEfSe* biomarkers with an LDA score of 4 or higher are presented. Genera in bold were previously associated with phenyl acid formation in a study using protein-rich substrates [21]

## Conclusions

Even though coming from a low carbon load system, the mesophilic sludge community could cope far better with lignin intermediates than the thermophilic one, especially at MCL conditions. Acetoclastic methanogens were shown to be especially susceptible to aromatic compound additions. HCL samples led to a complete inhibition of methanogenesis. Lignin intermediates also led to an inhibition of acetogenic microorganisms, especially at HCL conditions. Not only the load and the temperature regime, but also the chemical structure (like the length of the aliphatic side chain, methoxy-, hydroxy-groups) of the lignin intermediates had an influence on the overall digestion performance. The phenyl acids PAA, PPA, and PBA accumulated when lignin intermediates were metabolically utilised, but stuck in degradation phases prior to methanogenesis. Previously identified associations of genera like *Fastidiosipila*, *Syntrophomonas* (mesophilic), or *Bacillus*, *Lactobacillus*, *Geobacillus*, and *Tissierella* (thermophilic) with anaerobic phenyl acid formation were reproduced; hence, these genera can be seen as bioindicators for phenyl acid formation and/or process failures. Although the impact of various lignin intermediates on the generation of phenyl acids could be established, the exact degradation pathways remain elusive. Future work implementing pure cultures of adequate model organisms or at least co-cultures with a manageable number of microbial species, as well as using multi’omic approaches like metatranscriptomics or metabolomics [53] could pave the way towards more detailed studies of complex natural systems.

## Methods

### Experimental setup and sampling

The mesophilic inoculum was taken from a wastewater treatment plant in Zirl (Austria), which is operated at a temperature of 39 °C and was characterised by a volatile solids (VS) concentration of 2.22 ± 0.04% in fresh matter and by a pH of 7.4 [25]. The thermophilic inoculum derived from the outlet sampling port of a green- and biowaste treating, full-scale digestion plant (Roppen, Austria), which is characterised by an operation temperature of 53 °C, a DM content of 24.3 ± 2.80%, an OM content of 60.8 ± 5.33% in dry matter, and a pH of 8.5 [54, 55]. Additional information regarding digester capacities, conditions, and characteristics can be looked up elsewhere [12, 19, 25]. The respective sludge was filled in plastic bottles (20L) and subsequently brought to the laboratory. For liquid handling, the sludge was sieved and diluted as described previously [19, 56] except that the mesophilic sludge was diluted 1:2 (instead of 1:5). Headspace exchange, removal of residual VFAs, and sludge storage were done according earlier protocols [19].

To initiate phenyl acid formation in batch reactors, five different lignin intermediates in three different concentrations (LCL, MCL, and HCL, Table 1) were each mixed with an anaerobic medium and a mesophilic or thermophilic inoculum. The respective lignin intermediate in the respective concentration was filled in triplicates into 120 mL serum flasks equipped with butyl stoppers. A basic anaerobic medium-containing carboxymethylcellulose was prepared as described previously [19]. Subsequently, 48 mL of medium were filled in each batch reactor. Gas-tight sealing, headspace gas exchange, autoclaving, and inoculum addition (12 mL) were done as described before [12]. The mesophilic and thermophilic reactors were incubated at 37 °C and 52 °C for 28 days, respectively (2 treatments). A control containing the anaerobic medium but no additional substrate was also included and equally prepared and treated from the medium addition thenceforward. In total, the use of 5 different lignin intermediates (variable 1) in three different concentrations (variable 2), the inoculation with a meso- or thermophilic sludge (variable 3), the establishment of controls for each temperature regime, and the preparation of each variant in triplicates, resulted in studying 96 batch reactors. For biochemical analyses, samples were taken on day 0, 2, 4, 7, 14, 21, and 28. The pH of the samples was immediately measured with pH indicator strips pH 5.0–10.0 (pH resolution: 0.5, Merck, Germany). Thereafter, samples were frozen at − 20 °C until further use.

### Biochemical analyses

Gas (CH_4_, CO_2_, and H_2_) concentration measurements via GC-TCD and calculations were done as described before [56, 57]. The VFA (acetate, propionate, i-butyrate, and butyrate) and phenyl acid (PAA, PPA, 2-PBA, 3-PBA, 4-PBA, benzoate, hydroxybenzoate, and hydroxy-PAA) concentrations were analysed using HPLC–UV/Vis at 220 and 270 nm [12, 38]. The “phenyl acid (sum)” parameter represents the calculated sum of all phenyl acid concentrations of each sample (Figs. 1 and 4). The gas over-pressure was measured with a GHM Greisinger GDH 200 sensor and used to calculate biogas and methane production (NmL) as described previously [57].

### *DNA extraction*, *library preparations*, *and amplicon sequencing*

For DNA extraction, liquid samples were processed as described earlier [19] using the Soil Extract II Kit DNA (Macherey–Nagel) except that the procedure did not include the addition of the enhancer solution. This was done to avoid co-extraction of PCR-inhibitory (aromatic) substances. The extracts were eluted in 30 µL of elution buffer. The equality of the microbial community structure of start-up samples from day 0 (*n* = 48) was checked and confirmed according to previously established protocols [58, 59]. Thereafter, for each temperature regime, sequencing analyses were done for controls of day 0 (which represent the microbial communities of all samples on day 0), as well as for samples of day 14 (*n* = 48), and 28 (*n* = 48). The library for amplicon sequencing was prepared in-house as described earlier [19]: The small subunit rRNA gene primers 515f and 806r [60], targeting the V4 region, were applied. The first PCR step included the Illumina® adapter sequences. The quality of the PCR products was checked with a 1.5% agarose gel. For attaching the Illumina® barcodes (second PCR step), the PCR products were amplified for seven cycles. The products of the second PCR were qualitatively checked and fluorometrically quantified. Subsequently, 15 ng of each PCR product were pooled, purified, and eluted in 50 µL Tris–HCl buffer. The final ready-to-load sample pool showed a 260/280 absorbance ratio of 1.89 and was subsequently sent to Microsynth AG in Switzerland for sequencing.

### Reads procession and OTU classification

In total, 99 mesophilic, 99 thermophilic, as well as 4 MOCK community samples were analysed. Raw sample reads were processed using the program mothur version 1.39.5 and 1.42.1 [61]: a contig file was created with the paired-end reads [9,680,593 sequences in total (without MOCK), 48,892 ± 12,467 sequences sample^−1^]. Approximately 29% of the sequences were discarded after sequence screening. Unique sequences were aligned to the SILVA V132 database, which was processed as described earlier [19]. Chimeric amplicons were removed using the VSEARCH algorithm (v2.3.4) [62]. Sequences were classified via the k-nearest neighbor (knn) algorithm and subsequently binned to phylotypes based on their taxonomy. After rarefaction analyses, samples were normalised to 9554 reads per sample. The OTU matrices prior to and after subsampling did not differ significantly from each other (*Mantel* test; *R* = 0.92, *p* < 0.01, *N* = 9999).

The MOCK samples were co-processed with reactor samples in 4 replicates to validate the sequencing procedure. Each community contained the ZymoBIOMICS™ Microbial Community standard (Zymo, containing eight bacterial and two yeast microorganisms) and the archaeon *Methanosarcina thermophila* DSM 1825 (DSMZ, German Collection of Microorganisms and Cell Cultures). The MOCK samples were checked via Microsoft® Excel®. All microorganisms could be recovered at genus level. The validity and reliability of the applied strategies for DNA extraction, library preparation, and data processing were thus proven.

The obtained raw OTU tables can be acquired from the authors upon request. Quality-filtered sequences were uploaded to GenBank® via *BankIt*.

### Graphical and statistical analyses

After rarefaction analyses, meso- and thermophilic data were analysed separately. For each temperature regime, OTUs with a total abundance below 50 were excluded as they would have made the data set too noisy. Phenyl acid (sum) concentrations, measured throughout the experiment, were grouped by k-means clustering (low PAG: 0–3.98 mM, medium PAG: 4.98–9.11 mM, and high PAG: 13.7–21.1 mM) via the program PAST® 3 [63]. In mothur, the core microbiome and the *LEfSe* biomarkers [64] of each PAG (low, medium, and high) were assessed [59, 64]. The *Mantel* test and NMDS ordination (two-dimensional), both using the Gower Similarity Index, were conducted in PAST® 3. For NMDS analyses, the respective *Shepard* plots and stress values were checked. For statistical analyses, the *Kruskal–Wallis* H test (KW-H) was conducted in Statistica™ 13 for diversity analyses and in STAMP 2.1.3 [65] for extended error bar analyses (including *Welch*’*s* post hoc test (95%) and *Benjamini–Hochberg* corrections [66]). White’s non-parametric t test (two-sided, 95% confidence interval calculated via bootstrapping; *Benjamini–Hochberg* corrections) and the *Mann–Whitney* U test were used for two independent variables, and were conducted in STAMP 2.1.3 and Statistica™ 13, respectively. The significance cut-off was set at *α* = 0.05 for all analyses. Box plots and bar plots were conducted in Statistica™ 13 and SigmaPlot™ 14 (Systat® Software Inc.), respectively. Extended error bar plots were done with STAMP 2.1.3; an effect size > 1 was chosen for a better visualisation of genera showing remarkable differences in their abundance among variants. The diversity analyses (*Shannon–Weaver* index) were done with R-4.0.0. [67] using the packages *ggplot2* [68], *phyloseq* [69], and *extrafont* [70]. All R analyses were done in RStudio® [71]. For an interactive visualisation of relative sequence abundances of meso- and thermophilic samples, the tool KRONA was used [72]. The KRONA graphs can be looked up in Additional file 1.

### Prediction of metagenomic properties

*Piphillin* analyses [73] were conducted according to previous investigations [19]: Representative sequences and the OTU table were uploaded to https://piphillin.secondgenome.com in February 2020. The KEGG database [74] of October 2018 was applied. The analyses focused on general biochemical pathways such as carbon fixation pathways in prokaryotes (KEGG orthology ko00720), nitrogen metabolism (KEGG orthology ko00910), sulphur metabolism (KEGG orthology ko00920), and methane metabolism (KEGG orthology ko00680), as well as pathways regarding turnover of aromatic compounds such as degradation of aromatic compounds (KEGG orthology ko01220) and phenylpropanoid biosynthesis (KEGG orthology ko00940). The focus lied also on the enzyme glycin dehydrogenase (K00282 and K00283) and on the ABC-2 type transport system ATP-binding protein (K01990). The neighbour-joining clustering with KEGG enzymes was done via PAST® 4 [63]. The heatmap for the most abundant and most differently expressed enzymes was created with R-4.0.0. [67] using the packages *pheatmap* [75], *readxl *[76], and *extrafont* [70]. The *piphillin* results can be looked up in Additional file 1.

## Supplementary Information

**Additional file 1: Table S1.** pH and concentrations of propionate, i-butyrate, and butyrate of mesophilic control and lignin intermediate samples under low, medium, and high overload conditions on day 0, 7, 14, and 28. **Table S2.** Concentrations of PAA, PPA, and PBA (sum) of mesophilic control and lignin intermediate samples under low, medium, and high overload conditions on day 0, 7, 14, and 28. **Table S3.** Concentrations of benzoate, hydroxybenzoate, and hydroxy-PAA of mesophilic control and lignin intermediate samples under low, medium, and high overload conditions on day 0, 7, 14, and 28. **Table S4.** pH and concentrations of propionate, i-butyrate, and butyrate of thermophilic control and lignin intermediate samples under low, medium, and high overload conditions on day 0, 7, 14, and 28. **Table S5.** Concentrations of PAA, PPA, and PBA (sum) of thermophilic control and lignin intermediate samples under low, medium, and high overload conditions on day 0, 7, 14, and 28. **Table S6.** Concentrations of benzoate, hydroxybenzoate, and hydroxy-PAA of thermophilic control and lignin intermediate samples under low, medium, and high overload conditions on day 0, 7, 14, and 28. **Figure S1a.** Mean sequence proportions [%] of mesophilic genera of all control and gallic, syringic, and vanillic acid samples. **Figure S1b.** Mean sequence proportions [%] of mesophilic genera of all control and ferulic acid and of all control and coumaric acid samples**. Figure S2a.** Interactive visualisation of mesophilic taxa of the control and all lignin intermediate samples under low, medium, and high overload conditions on day 0 and on day 14. **Figure S2b.** Interactive visualisation of mesophilic taxa of the control and all lignin intermediate samples under low, medium, and high overload conditions on day 0 and on day 28. **Figure S3.** KEGG orthology counts of general pathways and of pathways relevant for AD of aromatic compounds of all mesophilic samples of day 28. **Figure S4a.** Mean sequence proportions [%] of thermophilic genera of all control and gallic, syringic, and vanillic acid samples. **Figure S4b.** Mean sequence proportions [%] of thermophilic genera of all control and ferulic acid and of all control and coumaric acid samples. **Figure S5a.** Interactive visualisation of thermophilic taxa of the control and of all lignin intermediates samples under low, medium, and high overload conditions on day 0 and on day 14. **Figure S5b**. Interactive visualisation of thermophilic taxa of the control and of all lignin intermediate samples under low, medium, and high overload conditions on day 0 and on day 28. **Figure S6.** KEGG orthology counts of general pathways and of pathways relevant for AD of aromatic compounds of all thermophilic samples on day 28. **Figure S7.** Shannon diversity index for meso- and thermophilic samples fed with gallic, syringic, vanillic, ferulic, and coumaric acid. **Figure S8a.** Neighbour joining clustering with KEGG enzyme results of thermophilic samples on day 28. **Figure S8b.** Heatmap using KEGG orthology counts of the most abundant and most differently expressed enzymes of thermophilic controls and HCL samples of day 28. **Figure S8c.** KEGG orthology counts per samples of the enzyme subunits K00282 (glycin dehydrogenase (gcvP) subunit 1) and K00283 (gcvP subunit 2) as well of K01990 (ABC-2 type transport system ATP-binding protein) of thermophilic controls and HCL samples of day 28. **Text S1.** *Piphillin* results. (DOCX 7742 kb)

## Data Availability

Mesophilic and thermophilic sequences were uploaded to GenBank® via the submission tool BankIt (BioProject ID for mesophilic samples: PRJNA606421, BioProject ID for thermophilic samples: PRJNA606449). Raw OTU tables can be acquired from the authors upon request.

## Abbreviations

AD: Anaerobic digestion
PAA: Phenylacetate
PPA: Phenylpropionate
PBA: Phenylbutyrate
VFA: Volatile fatty acid
LCL: Low carbon load
cum: Cumulative
MCL: Medium carbon load
HCL: High carbon load
OTU: Operational taxonomic unit
NMDS: Non-metric multidimensional scaling
*LEfSe*: Linear discriminant analysis effect size
PAG: Phenyl acid group
SAOB: Syntrophic acetate oxidising bacterium
SAO: Syntrophic acetate oxidation
KEGG: Kyoto Encyclopedia of Genes and Genomes
knn: K-nearest neighbor
Zymo: ZymoBIOMICS™ Microbial Community standard
DSMZ: German Collection of Microorganisms and Cell Cultures
KW-H: *Kruskal–Wallis* H test
LDA: Linear discriminant analysis
