# Supplementary material for: Lignin intermediates lead to phenyl acid formation and microbial community shifts in meso- and thermophilic batch reactors
Source: Biotechnol Biofuels. 2021 Jan 20;14:27. doi: 10.1186/s13068-020-01855-0 (PMC7816434; doi:10.1186/s13068-020-01855-0)
Supplement: Supplementary file 1 — Additional file 1: Table S1. pH and concentrations of propionate, i-butyrate, and butyrate of mesophilic control and lignin intermediate samples under low, medium, and high overload conditions on day 0, 7, 14, and 28. Table S2. Concentrations of PAA, PPA, and PBA (sum) of mesophilic control and lignin intermediate samples under low, medium, and high overload conditions on day 0, 7, 14, and 28. Table S3. Concentrations of benzoate, hydroxybenzoate, and hydroxy-PAA of mesophilic control and lignin intermediate samples under low, medium, and high overload conditions on day 0, 7, 14, and 28. Table S4. pH and concentrations of propionate, i-butyrate, and butyrate of thermophilic control and lignin intermediate samples under low, medium, and high overload conditions on day 0, 7, 14, and 28. Table S5. Concentrations of PAA, PPA, and PBA (sum) of thermophilic control and lignin intermediate samples under low, medium, and high overload conditions on day 0, 7, 14, and 28. Table S6. Concentrations of benzoate, hydroxybenzoate, and hydroxy-PAA of thermophilic control and lignin intermediate samples under low, medium, and high overload conditions on day 0, 7, 14, and 28. Figure S1a. Mean sequence proportions [%] of mesophilic genera of all control and gallic, syringic, and vanillic acid samples. Figure S1b. Mean sequence proportions [%] of mesophilic genera of all control and ferulic acid and of all control and coumaric acid samples. Figure S2a. Interactive visualisation of mesophilic taxa of the control and all lignin intermediate samples under low, medium, and high overload conditions on day 0 and on day 14. Figure S2b. Interactive visualisation of mesophilic taxa of the control and all lignin intermediate samples under low, medium, and high overload conditions on day 0 and on day 28. Figure S3. KEGG orthology counts of general pathways and of pathways relevant for AD of aromatic compounds of all mesophilic samples of day 28. Figure S4a. Mean sequence proportions [%] of thermo [file 13068_2020_1855_MOESM1_ESM.docx]

Type of contribution: Supplementary

Number of figures: 8

Number of tables: 6

Title

Lignin Intermediates Lead to Phenyl Acid Formation and Microbial Community Shifts in Meso- and Thermophilic Batch Reactors

Authors:

Eva Maria Prem^1*^, Mira Mutschlechner^1^, Blaz Stres^2,3,4^, Paul Illmer^1^, and Andreas Otto Wagner^1^

Affiliation:

^1^Department of Microbiology, Universität Innsbruck, Technikerstr. 25d, 6020 Innsbruck, Austria

^2^Department of Animal Science, Biotechnical Faculty, University of Ljubljana, Jamnikarjeva 101, SI-1000 Ljubljana, Slovenia

^3^Institute of Sanitary Engineering, Faculty of Civil and Geodetic Engineering, University of Ljubljana, Jamova 2, SI-1000 Ljubljana, Slovenia

^4^Department of Automation, Biocybernetics and Robotics, Jozef Štefan Institute, Jamova 39, SI-1000 Ljubljana, Slovenia

^*^Corresponding author:

Eva Maria Prem, Department of Microbiology, Universität Innsbruck, Technikerstraße 25d, 6020 Innsbruck, Austria, Tel.: 0043 512 507 51346, email: [eva.prem@uibk.ac.at](mailto:eva.prem@uibk.ac.at)

1. Supplementary Results

Table S1: pH values and concentrations of propionate, i-butyrate, and butyrate of mesophilic control samples as well as of mesophilic samples fed with the respective lignin intermediate (gallic, syringic, vanillic, ferulic, or coumaric acid) under low, medium, and high overload conditions on day 0, 7, 14, and 28 . VFA results are shown in mM; mean values are presented with the respective standard deviations (SD).

| **Day** | **Source** | **Load** | **pH** | | **Propionate** | | **i-Butyrate** | | **Butyrate** | | **Day** | **Source** | **Load** | **pH** | | **Propionate** | | **i-Butyrate** | | **Butyrate** | |
| --- | --- | --- | --- | --- | --- | --- | --- | --- | --- | --- | --- | --- | --- | --- | --- | --- | --- | --- | --- | --- | --- |
|  |  |  | **mean** | **SD** | **mean** | **SD** | **mean** | **SD** | **mean** | **SD** |  |  |  | **mean** | **SD** | **mean** | **SD** | **mean** | **SD** | **mean** | **SD** |
| 0 | control | control | 7.00 | 0.00 | 0.20 | 0.34 | 0.38 | 0.03 | 0.00 | 0.00 | 14 | control | control | 7.00 | 0.00 | 2.66 | 0.88 | 0.34 | 0.14 | 1.33 | 0.19 |
|  | gallic acid | low | 7.00 | 0.00 | 0.65 | 0.04 | 0.53 | 0.02 | 0.00 | 0.00 |  | gallic acid | low | 7.00 | 0.00 | 2.12 | 0.46 | 0.42 | 0.32 | 1.40 | 0.26 |
|  |  | medium | 7.00 | 0.00 | 0.48 | 0.17 | 1.21 | 0.72 | 0.00 | 0.00 |  |  | medium | 6.50 | 0.00 | 1.51 | 0.47 | 0.26 | 0.03 | 1.96 | 0.30 |
|  |  | high | 7.00 | 0.00 | 1.26 | 0.48 | 0.00 | 0.00 | 0.00 | 0.00 |  |  | high | 5.50 | 0.00 | 8.28 | 3.51 | 0.00 | 0.00 | 0.00 | 0.00 |
|  | syringic acid | low | 7.00 | 0.00 | 0.38 | 0.17 | 0.38 | 0.15 | 0.00 | 0.00 |  | syringic acid | low | 7.00 | 0.00 | 3.12 | 0.63 | 0.29 | 0.09 | 1.45 | 0.16 |
|  |  | medium | 7.00 | 0.00 | 0.64 | 0.02 | 0.49 | 0.05 | 0.00 | 0.00 |  |  | medium | 7.00 | 0.00 | 3.03 | 0.89 | 0.22 | 0.05 | 4.86 | 0.15 |
|  |  | high | 7.00 | 0.00 | 0.59 | 0.25 | 0.30 | 0.02 | 0.00 | 0.00 |  |  | high | 5.00 | 0.00 | 2.46 | 1.22 | 4.64 | 0.11 | 0.87 | 0.29 |
|  | vanillic acid | low | 7.00 | 0.00 | 0.62 | 0.04 | 0.47 | 0.03 | 0.00 | 0.00 |  | vanillic acid | low | 7.00 | 0.00 | 2.82 | 0.62 | 0.26 | 0.05 | 1.46 | 0.06 |
|  |  | medium | 7.00 | 0.00 | 0.59 | 0.05 | 0.35 | 0.02 | 0.00 | 0.00 |  |  | medium | 7.00 | 0.00 | 1.16 | 0.11 | 0.59 | 0.59 | 3.41 | 0.16 |
|  |  | high | 7.00 | 0.00 | 0.68 | 0.03 | 0.29 | 0.09 | 0.00 | 0.00 |  |  | high | 5.00 | 0.00 | 0.46 | 0.02 | 4.59 | 0.44 | 0.47 | 0.21 |
|  | ferulic acid | low | 7.00 | 0.00 | 0.64 | 0.03 | 0.47 | 0.01 | 0.00 | 0.00 |  | ferulic acid | low | 7.00 | 0.00 | 2.57 | 0.51 | 0.64 | 0.22 | 1.53 | 0.14 |
|  |  | medium | 7.00 | 0.00 | 0.75 | 0.07 | 0.38 | 0.03 | 0.00 | 0.00 |  |  | medium | 6.50 | 0.00 | 3.24 | 0.15 | 0.75 | 0.69 | 1.57 | 0.37 |
|  |  | high | 7.00 | 0.00 | 0.83 | 0.01 | 0.24 | 0.02 | 0.00 | 0.00 |  |  | high | 5.50 | 0.00 | 0.32 | 0.36 | 4.46 | 0.26 | 0.00 | 0.00 |
|  | coumaric acid | low | 7.00 | 0.00 | 0.72 | 0.06 | 0.41 | 0.04 | 0.00 | 0.00 |  | coumaric acid | low | 7.00 | 0.00 | 4.48 | 1.64 | 0.76 | 0.31 | 1.40 | 0.05 |
|  |  | medium | 7.00 | 0.00 | 0.69 | 0.01 | 0.42 | 0.01 | 0.00 | 0.00 |  |  | medium | 7.00 | 0.00 | 2.98 | 0.33 | 0.37 | 0.04 | 4.06 | 0.59 |
|  |  | high | 7.00 | 0.00 | 0.68 | 0.02 | 0.25 | 0.02 | 0.00 | 0.00 |  |  | high | 5.00 | 0.00 | 0.49 | 0.29 | 4.57 | 0.17 | 9.99 | 0.59 |
| 7 | control | control | 7.50 | 0.00 | 1.43 | 0.16 | 0.09 | 0.02 | 0.30 | 0.07 | 28 | control | control | 7.50 | 0.00 | 3.12 | 0.85 | 0.11 | 0.12 | 0.00 | 0.00 |
|  | gallic acid | low | 7.00 | 0.00 | 1.63 | 0.04 | 0.10 | 0.01 | 0.35 | 0.03 |  | gallic acid | low | 7.00 | 0.00 | 1.91 | 0.91 | 0.10 | 0.17 | 0.15 | 0.25 |
|  |  | medium | 6.00 | 0.00 | 1.44 | 0.05 | 0.11 | 0.01 | 0.59 | 0.05 |  |  | medium | 7.00 | 0.00 | 0.36 | 0.08 | 0.00 | 0.00 | 1.60 | 0.82 |
|  |  | high | 5.50 | 0.00 | 2.32 | 0.32 | 8.93 | 2.57 | 8.68 | 5.25 |  |  | high | 5.50 | 0.00 | 2.16 | 0.45 | 0.00 | 0.00 | 0.00 | 0.00 |
|  | syringic acid | low | 7.00 | 0.00 | 1.36 | 0.23 | 0.11 | 0.00 | 0.48 | 0.08 |  | syringic acid | low | 7.00 | 0.00 | 2.65 | 1.30 | 0.16 | 0.14 | 0.00 | 0.00 |
|  |  | medium | 6.50 | 0.00 | 1.56 | 0.12 | 0.10 | 0.02 | 4.28 | 0.03 |  |  | medium | 7.00 | 0.00 | 0.76 | 0.11 | 0.00 | 0.00 | 4.17 | 0.14 |
|  |  | high | 5.00 | 0.00 | 0.61 | 0.04 | 1.91 | 1.51 | 0.00 | 0.00 |  |  | high | 5.00 | 0.00 | 0.68 | 0.45 | 4.08 | 0.89 | 0.44 | 0.39 |
|  | vanillic acid | low | 7.00 | 0.00 | 1.72 | 0.03 | 0.11 | 0.01 | 0.37 | 0.03 |  | vanillic acid | low | 7.00 | 0.00 | 2.47 | 0.67 | 0.05 | 0.09 | 0.00 | 0.00 |
|  |  | medium | 7.00 | 0.00 | 1.16 | 0.15 | 0.02 | 0.03 | 2.13 | 0.06 |  |  | medium | 7.00 | 0.00 | 3.08 | 0.32 | 0.00 | 0.00 | 2.02 | 0.23 |
|  |  | high | 5.00 | 0.00 | 0.63 | 0.01 | 0.63 | 0.11 | 0.00 | 0.00 |  |  | high | 5.00 | 0.00 | 0.49 | 0.03 | 4.56 | 0.48 | 0.15 | 0.13 |
|  | ferulic acid | low | 7.00 | 0.00 | 1.14 | 0.20 | 0.60 | 0.13 | 0.34 | 0.05 |  | ferulic acid | low | 7.00 | 0.00 | 4.33 | 0.42 | 0.23 | 0.40 | 0.00 | 0.00 |
|  |  | medium | 6.50 | 0.00 | 2.13 | 1.32 | 1.90 | 0.34 | 0.63 | 0.24 |  |  | medium | 7.00 | 0.00 | 6.71 | 4.65 | 2.15 | 0.35 | 0.70 | 0.33 |
|  |  | high | 5.50 | 0.00 | 1.09 | 0.03 | 0.71 | 0.09 | 0.00 | 0.00 |  |  | high | 5.50 | 0.00 | 0.34 | 0.30 | 4.53 | 0.58 | 0.00 | 0.00 |
|  | coumaric acid | low | 7.00 | 0.00 | 1.64 | 0.16 | 0.08 | 0.02 | 0.53 | 0.15 |  | coumaric acid | low | 7.50 | 0.00 | 2.79 | 0.90 | 0.43 | 0.17 | 0.84 | 0.17 |
|  |  | medium | 7.00 | 0.00 | 0.85 | 0.01 | 0.12 | 0.03 | 1.95 | 0.24 |  |  | medium | 6.50 | 0.00 | 2.36 | 0.32 | 0.21 | 0.04 | 2.39 | 0.22 |
|  |  | high | 5.00 | 0.00 | 0.59 | 0.02 | 0.48 | 0.01 | 2.18 | 0.54 |  |  | high | 5.00 | 0.00 | 0.49 | 0.02 | 4.26 | 0.08 | 8.32 | 0.27 |

Table S2: Concentrations of phenylacetate (PAA), phenylpropionate (PPA), and phenylbutyrate (PBA, sum) of mesophilic control samples as well as of mesophilic samples fed with the respective lignin intermediate (gallic, syringic, vanillic, ferulic, or coumaric acid) under low, medium, and high overload conditions on day 0, 7, 14, and 28 . “PBA sum” represents the sum of the 2-PBA, 3-PBA, and 4-PBA concentrations of each variation. Results are shown in mM. Mean values are presented with the respective standard deviations (SD).

| **Day** | **Source** | **Load** | **PAA** | | **PPA** | | **PBA (sum)** | | **Day** | **Source** | **Load** | **PAA** | | **PPA** | | **PBA (sum)** | |
| --- | --- | --- | --- | --- | --- | --- | --- | --- | --- | --- | --- | --- | --- | --- | --- | --- | --- |
|  |  |  | **mean** | **SD** | **mean** | **SD** | **mean** | **SD** |  |  |  | **mean** | **SD** | **mean** | **SD** | **mean** | **SD** |
| 0 | control | control | 0.00 | 0.00 | 0.00 | 0.00 | 0.00 | 0.00 | 14 | control | control | 0.20 | 0.01 | 0.09 | 0.02 | 0.04 | 0.05 |
|  | gallic acid | low | 0.00 | 0.00 | 0.00 | 0.00 | 0.00 | 0.00 |  | gallic acid | low | 0.21 | 0.02 | 0.09 | 0.02 | 0.03 | 0.05 |
|  |  | medium | 0.00 | 0.00 | 0.00 | 0.00 | 0.00 | 0.00 |  |  | medium | 0.22 | 0.03 | 0.08 | 0.01 | 0.00 | 0.00 |
|  |  | high | 0.00 | 0.00 | 0.00 | 0.00 | 0.00 | 0.00 |  |  | high | 1.76 | 0.21 | 0.01 | 0.01 | 0.00 | 0.00 |
|  | syringic acid | low | 0.00 | 0.00 | 0.00 | 0.00 | 0.00 | 0.00 |  | syringic acid | low | 0.22 | 0.02 | 0.07 | 0.02 | 0.01 | 0.00 |
|  |  | medium | 0.00 | 0.00 | 0.00 | 0.00 | 0.00 | 0.01 |  |  | medium | 0.25 | 0.03 | 0.07 | 0.01 | 0.00 | 0.00 |
|  |  | high | 0.00 | 0.00 | 0.00 | 0.00 | 0.06 | 0.00 |  |  | high | 0.21 | 0.01 | 0.01 | 0.02 | 0.11 | 0.09 |
|  | vanillic acid | low | 0.00 | 0.00 | 0.00 | 0.00 | 0.00 | 0.00 |  | vanillic acid | low | 0.02 | 0.00 | 0.09 | 0.00 | 0.00 | 0.00 |
|  |  | medium | 0.00 | 0.00 | 0.00 | 0.00 | 0.00 | 0.00 |  |  | medium | 0.02 | 0.00 | 0.09 | 0.01 | 0.00 | 0.00 |
|  |  | high | 0.00 | 0.00 | 0.00 | 0.00 | 0.00 | 0.00 |  |  | high | 0.02 | 0.00 | 0.32 | 0.56 | 5.01 | 1.77 |
|  | ferulic acid | low | 0.00 | 0.00 | 0.00 | 0.00 | 0.63 | 0.00 |  | ferulic acid | low | 0.03 | 0.01 | 0.48 | 0.02 | 0.00 | 0.00 |
|  |  | medium | 0.00 | 0.00 | 0.00 | 0.00 | 0.01 | 0.00 |  |  | medium | 0.04 | 0.02 | 0.31 | 0.08 | 0.00 | 0.00 |
|  |  | high | 0.00 | 0.00 | 0.00 | 0.00 | 0.00 | 0.00 |  |  | high | 0.00 | 0.00 | 0.00 | 0.00 | 0.17 | 0.01 |
|  | coumaric acid | low | 0.00 | 0.00 | 0.00 | 0.00 | 0.05 | 0.00 |  | coumaric acid | low | 0.22 | 0.19 | 0.54 | 0.06 | 0.00 | 0.00 |
|  |  | medium | 0.18 | 0.01 | 0.00 | 0.00 | 0.16 | 0.00 |  |  | medium | 1.82 | 0.11 | 5.44 | 0.09 | 0.24 | 0.06 |
|  |  | high | 0.42 | 0.05 | 0.00 | 0.00 | 0.15 | 0.00 |  |  | high | 14.6 | 1.69 | 0.32 | 0.04 | 0.27 | 0.03 |
| 7 | control | control | 0.01 | 0.01 | 0.00 | 0.00 | 0.00 | 0.00 | 28 | control | control | 0.21 | 0.01 | 0.03 | 0.03 | 0.06 | 0.06 |
|  | gallic acid | low | 0.00 | 0.00 | 0.00 | 0.00 | 0.00 | 0.00 |  | gallic acid | low | 0.23 | 0.01 | 0.05 | 0.01 | 0.05 | 0.06 |
|  |  | medium | 0.00 | 0.01 | 0.00 | 0.00 | 0.00 | 0.00 |  |  | medium | 0.26 | 0.02 | 0.08 | 0.01 | 0.00 | 0.00 |
|  |  | high | 0.36 | 0.62 | 0.00 | 0.00 | 0.00 | 0.00 |  |  | high | 0.52 | 0.49 | 0.01 | 0.02 | 0.01 | 0.01 |
|  | syringic acid | low | 0.01 | 0.01 | 0.00 | 0.00 | 0.00 | 0.00 |  | syringic acid | low | 0.25 | 0.01 | 0.01 | 0.02 | 0.33 | 0.26 |
|  |  | medium | 0.00 | 0.00 | 0.00 | 0.00 | 0.00 | 0.00 |  |  | medium | 0.26 | 0.02 | 0.06 | 0.01 | 0.03 | 0.03 |
|  |  | high | 0.00 | 0.00 | 0.00 | 0.00 | 0.06 | 0.00 |  |  | high | 0.23 | 0.01 | 0.01 | 0.02 | 0.06 | 0.00 |
|  | vanillic acid | low | 0.00 | 0.00 | 0.00 | 0.00 | 0.00 | 0.00 |  | vanillic acid | low | 0.26 | 0.02 | 0.00 | 0.01 | 0.45 | 0.13 |
|  |  | medium | 0.00 | 0.00 | 0.00 | 0.00 | 0.00 | 0.00 |  |  | medium | 0.23 | 0.01 | 0.08 | 0.00 | 0.00 | 0.01 |
|  |  | high | 0.00 | 0.00 | 0.00 | 0.00 | 2.30 | 2.17 |  |  | high | 0.16 | 0.14 | 1.54 | 0.02 | 3.31 | 1.99 |
|  | ferulic acid | low | 0.02 | 0.02 | 0.00 | 0.00 | 0.00 | 0.00 |  | ferulic acid | low | 0.28 | 0.01 | 0.00 | 0.00 | 1.82 | 0.04 |
|  |  | medium | 0.58 | 0.48 | 0.00 | 0.00 | 0.02 | 0.03 |  |  | medium | 1.13 | 0.68 | 0.80 | 0.15 | 0.13 | 0.12 |
|  |  | high | 0.00 | 0.00 | 0.00 | 0.00 | 0.15 | 0.01 |  |  | high | 0.00 | 0.00 | 0.00 | 0.00 | 0.19 | 0.02 |
|  | coumaric acid | low | 0.00 | 0.00 | 0.00 | 0.00 | 0.00 | 0.00 |  | coumaric acid | low | 0.34 | 0.01 | 0.83 | 0.18 | 0.01 | 0.02 |
|  |  | medium | 0.18 | 0.01 | 0.62 | 0.53 | 0.19 | 0.08 |  |  | medium | 0.86 | 0.15 | 7.22 | 0.11 | 0.57 | 0.39 |
|  |  | high | 1.20 | 1.14 | 0.00 | 0.00 | 0.25 | 0.03 |  |  | high | 17.9 | 0.66 | 0.87 | 0.58 | 0.28 | 0.04 |

Table S3: Concentrations of benzoate, hydroxybenzoate, and hydroxy-PAA of mesophilic control samples as well as of mesophilic samples fed with the respective lignin intermediate (gallic, syringic, vanillic, ferulic, or coumaric acid) under low, medium, and high overload conditions on day 0, 7, 14, and 28 . Results are shown mM. Mean values are presented with the respective standard deviations (SD).

| **Day** | **Source** | **Load** | **Benzoate** | | **Hydroxy-benzoate** | | **Hydroxy-PAA** | | **Day** | **Source** | **Load** | **Benzoate** | | **Hydroxy-benzoate** | | **Hydroxy-PAA** | |
| --- | --- | --- | --- | --- | --- | --- | --- | --- | --- | --- | --- | --- | --- | --- | --- | --- | --- |
|  |  |  | **mean** | **SD** | **mean** | **SD** | **mean** | **SD** |  |  |  | **mean** | **SD** | **mean** | **SD** | **mean** | **SD** |
| 0 | control | control | 0.00 | 0.00 | 0.00 | 0.00 | 0.00 | 0.00 | 14 | control | control | 0.00 | 0.00 | 0.00 | 0.00 | 0.00 | 0.00 |
|  | gallic acid | low | 0.00 | 0.00 | 0.00 | 0.00 | 0.00 | 0.00 |  | gallic acid | low | 0.00 | 0.00 | 0.00 | 0.00 | 0.00 | 0.00 |
|  |  | medium | 0.00 | 0.00 | 0.00 | 0.00 | 0.00 | 0.00 |  |  | medium | 0.00 | 0.00 | 0.00 | 0.00 | 0.00 | 0.00 |
|  |  | high | 0.00 | 0.00 | 0.00 | 0.00 | 0.00 | 0.00 |  |  | high | 0.00 | 0.00 | 0.00 | 0.00 | 0.03 | 0.00 |
|  | syringic acid | low | 0.00 | 0.00 | 0.00 | 0.00 | 0.00 | 0.00 |  | syringic acid | low | 0.00 | 0.00 | 0.00 | 0.00 | 0.00 | 0.00 |
|  |  | medium | 0.00 | 0.00 | 0.00 | 0.00 | 0.00 | 0.00 |  |  | medium | 0.00 | 0.00 | 0.00 | 0.00 | 0.00 | 0.00 |
|  |  | high | 0.00 | 0.00 | 0.00 | 0.00 | 0.02 | 0.03 |  |  | high | 0.00 | 0.00 | 0.17 | 0.01 | 0.00 | 0.00 |
|  | vanillic acid | low | 0.00 | 0.00 | 0.00 | 0.00 | 0.00 | 0.00 |  | vanillic acid | low | 0.00 | 0.00 | 0.05 | 0.01 | 0.11 | 0.00 |
|  |  | medium | 0.00 | 0.00 | 0.00 | 0.00 | 0.00 | 0.00 |  |  | medium | 0.00 | 0.00 | 0.03 | 0.01 | 1.66 | 0.11 |
|  |  | high | 0.00 | 0.00 | 0.00 | 0.00 | 0.00 | 0.00 |  |  | high | 0.00 | 0.00 | 0.08 | 0.01 | 0.00 | 0.00 |
|  | ferulic acid | low | 0.00 | 0.00 | 0.00 | 0.00 | 0.00 | 0.00 |  | ferulic acid | low | 0.00 | 0.00 | 0.01 | 0.00 | 0.00 | 0.00 |
|  |  | medium | 0.00 | 0.00 | 0.00 | 0.00 | 0.00 | 0.00 |  |  | medium | 0.01 | 0.01 | 0.00 | 0.00 | 0.05 | 0.06 |
|  |  | high | 0.00 | 0.00 | 0.00 | 0.00 | 0.00 | 0.01 |  |  | high | 0.00 | 0.00 | 0.43 | 0.38 | 0.05 | 0.03 |
|  | coumaric acid | low | 0.00 | 0.00 | 0.00 | 0.00 | 0.00 | 0.00 |  | coumaric acid | low | 0.00 | 0.00 | 0.00 | 0.00 | 0.00 | 0.00 |
|  |  | medium | 0.00 | 0.00 | 0.00 | 0.00 | 0.00 | 0.00 |  |  | medium | 0.00 | 0.00 | 0.00 | 0.00 | 0.00 | 0.00 |
|  |  | high | 0.00 | 0.00 | 0.00 | 0.00 | 0.00 | 0.00 |  |  | high | 0.00 | 0.00 | 0.00 | 0.00 | 0.00 | 0.00 |
| 7 | control | control | 0.00 | 0.00 | 0.00 | 0.00 | 0.00 | 0.00 | 28 | control | control | 0.00 | 0.00 | 0.00 | 0.00 | 0.00 | 0.00 |
|  | gallic acid | low | 0.00 | 0.00 | 0.00 | 0.00 | 0.00 | 0.00 |  | gallic acid | low | 0.00 | 0.00 | 0.00 | 0.00 | 0.00 | 0.00 |
|  |  | medium | 0.00 | 0.00 | 0.00 | 0.00 | 0.00 | 0.00 |  |  | medium | 0.00 | 0.00 | 0.00 | 0.00 | 0.00 | 0.00 |
|  |  | high | 0.00 | 0.00 | 0.00 | 0.00 | 0.42 | 0.04 |  |  | high | 0.00 | 0.00 | 0.00 | 0.00 | 0.00 | 0.00 |
|  | syringic acid | low | 0.00 | 0.00 | 0.00 | 0.00 | 0.00 | 0.00 |  | syringic acid | low | 0.00 | 0.00 | 0.00 | 0.00 | 0.00 | 0.00 |
|  |  | medium | 0.00 | 0.00 | 0.00 | 0.00 | 0.00 | 0.00 |  |  | medium | 0.00 | 0.00 | 0.00 | 0.00 | 0.00 | 0.00 |
|  |  | high | 0.00 | 0.00 | 0.17 | 0.00 | 0.00 | 0.00 |  |  | high | 0.00 | 0.00 | 0.22 | 0.02 | 0.00 | 0.00 |
|  | vanillic acid | low | 0.00 | 0.00 | 0.00 | 0.00 | 0.21 | 0.01 |  | vanillic acid | low | 0.00 | 0.00 | 0.00 | 0.00 | 0.00 | 0.00 |
|  |  | medium | 0.00 | 0.00 | 0.00 | 0.00 | 2.15 | 0.04 |  |  | medium | 0.00 | 0.00 | 1.25 | 0.29 | 1.65 | 0.16 |
|  |  | high | 0.00 | 0.00 | 0.07 | 0.01 | 0.00 | 0.00 |  |  | high | 0.00 | 0.00 | 0.07 | 0.02 | 0.00 | 0.00 |
|  | ferulic acid | low | 0.00 | 0.00 | 0.00 | 0.00 | 0.10 | 0.01 |  | ferulic acid | low | 0.00 | 0.00 | 0.00 | 0.00 | 0.00 | 0.00 |
|  |  | medium | 0.08 | 0.03 | 0.29 | 0.29 | 0.76 | 0.21 |  |  | medium | 0.00 | 0.00 | 0.00 | 0.00 | 0.05 | 0.05 |
|  |  | high | 0.00 | 0.00 | 0.00 | 0.00 | 0.01 | 0.01 |  |  | high | 0.00 | 0.00 | 0.00 | 0.00 | 0.04 | 0.01 |
|  | coumaric acid | low | 0.00 | 0.00 | 0.00 | 0.00 | 0.00 | 0.00 |  | coumaric acid | low | 0.00 | 0.00 | 0.00 | 0.00 | 0.00 | 0.00 |
|  |  | medium | 0.00 | 0.00 | 0.00 | 0.00 | 0.00 | 0.00 |  |  | medium | 0.00 | 0.00 | 0.00 | 0.00 | 0.00 | 0.00 |
|  |  | high | 0.00 | 0.00 | 0.00 | 0.00 | 0.00 | 0.00 |  |  | high | 0.00 | 0.00 | 0.00 | 0.00 | 0.00 | 0.00 |

Table S4: pH values and concentrations of propionate, i-butyrate, and butyrate of thermophilic control samples as well as of thermophilic samples fed with the respective lignin intermediate (gallic, syringic, vanillic, ferulic, or coumaric acid) under low, medium, and high overload conditions on day 0, 7, 14, and 28 . VFA results are shown in mM; mean values are presented with the respective standard deviations (SD).

| **Day** | **Source** | **Load** | **pH** | | **Propionate** | | **i-Butyrate** | | **Butyrate** | | **Day** | **Source** | **Load** | **pH** | | **Propionate** | | **i-Butyrate** | | **Butyrate** | |
| --- | --- | --- | --- | --- | --- | --- | --- | --- | --- | --- | --- | --- | --- | --- | --- | --- | --- | --- | --- | --- | --- |
|  |  |  | **mean** | **SD** | **mean** | **SD** | **mean** | **SD** | **mean** | **SD** |  |  |  | **mean** | **SD** | **mean** | **SD** | **mean** | **SD** | **mean** | **SD** |
| 0 | control | control | 7.00 | 0.00 | 0.00 | 0.00 | 0.00 | 0.00 | 1.68 | 0.00 | 14 | control | control | 7.50 | 0.00 | 2.67 | 0.88 | 1.34 | 0.19 | 0.13 | 0.02 |
|  | gallic acid | low | 7.00 | 0.00 | 0.00 | 0.00 | 0.00 | 0.00 | 1.82 | 0.02 |  | gallic acid | low | 7.00 | 0.00 | 2.13 | 0.47 | 1.40 | 0.26 | 0.28 | 0.05 |
|  |  | medium | 7.00 | 0.00 | 0.00 | 0.00 | 0.00 | 0.00 | 1.97 | 0.02 |  |  | medium | 6.00 | 0.00 | 1.52 | 0.47 | 1.97 | 0.30 | 0.39 | 0.06 |
|  |  | high | 7.00 | 0.00 | 0.00 | 0.00 | 0.00 | 0.00 | 2.02 | 0.10 |  |  | high | 5.00 | 0.00 | 2.00 | 0.15 | 0.00 | 0.00 | 0.97 | 0.15 |
|  | syringic acid | low | 7.00 | 0.00 | 0.00 | 0.00 | 0.00 | 0.00 | 1.82 | 0.02 |  | syringic acid | low | 7.00 | 0.00 | 3.13 | 0.64 | 1.45 | 0.16 | 0.14 | 0.02 |
|  |  | medium | 7.00 | 0.00 | 0.00 | 0.00 | 0.00 | 0.00 | 2.00 | 0.01 |  |  | medium | 6.50 | 0.00 | 3.05 | 0.89 | 0.49 | 0.01 | 4.88 | 0.15 |
|  |  | high | 7.00 | 0.00 | 1.63 | 0.05 | 0.00 | 0.00 | 2.47 | 0.02 |  |  | high | 5.00 | 0.00 | 2.47 | 1.22 | 0.87 | 0.29 | 0.09 | 0.03 |
|  | vanillic acid | low | 7.00 | 0.00 | 0.00 | 0.00 | 0.00 | 0.00 | 1.49 | 0.01 |  | vanillic acid | low | 7.00 | 0.00 | 2.87 | 0.64 | 1.45 | 0.07 | 0.15 | 0.01 |
|  |  | medium | 7.00 | 0.00 | 0.00 | 0.00 | 0.00 | 0.00 | 1.62 | 0.01 |  |  | medium | 7.00 | 0.00 | 1.18 | 0.11 | 3.40 | 0.17 | 3.41 | 0.16 |
|  |  | high | 7.00 | 0.00 | 2.12 | 0.03 | 0.00 | 0.00 | 2.38 | 0.02 |  |  | high | 5.00 | 0.00 | 0.46 | 0.03 | 0.47 | 0.21 | 0.05 | 0.02 |
|  | ferulic acid | low | 7.00 | 0.00 | 0.00 | 0.00 | 0.00 | 0.00 | 1.61 | 0.01 |  | ferulic acid | low | 7.00 | 0.00 | 2.61 | 0.52 | 1.52 | 0.14 | 0.15 | 0.01 |
|  |  | medium | 7.00 | 0.00 | 0.00 | 0.00 | 0.00 | 0.00 | 1.90 | 0.03 |  |  | medium | 6.50 | 0.00 | 3.28 | 0.16 | 1.56 | 0.37 | 0.16 | 0.04 |
|  |  | high | 7.00 | 0.00 | 0.00 | 0.00 | 0.00 | 0.00 | 2.26 | 0.02 |  |  | high | 5.00 | 0.00 | 0.32 | 0.36 | 0.00 | 0.00 | 0.00 | 0.00 |
|  | coumaric acid | low | 7.00 | 0.00 | 0.00 | 0.00 | 0.00 | 0.00 | 1.41 | 0.01 |  | coumaric acid | low | 7.00 | 0.00 | 4.48 | 1.64 | 1.40 | 0.05 | 0.84 | 0.03 |
|  |  | medium | 7.00 | 0.00 | 0.00 | 0.00 | 0.00 | 0.00 | 1.40 | 0.00 |  |  | medium | 7.00 | 0.00 | 2.98 | 0.33 | 4.06 | 0.59 | 2.44 | 0.35 |
|  |  | high | 7.00 | 0.00 | 2.15 | 0.04 | 0.00 | 0.00 | 1.70 | 0.01 |  |  | high | 5.00 | 0.00 | 0.49 | 0.29 | 9.99 | 0.59 | 5.99 | 0.36 |
| 7 | control | control | 7.50 | 0.00 | 1.64 | 0.10 | 0.00 | 0.00 | 0.26 | 0.04 | 28 | control | control | 7.50 | 0.00 | 3.16 | 0.86 | 0.10 | 0.11 | 0.00 | 0.00 |
|  | gallic acid | low | 7.00 | 0.00 | 1.80 | 0.05 | 0.00 | 0.00 | 0.59 | 0.08 |  | gallic acid | low | 7.00 | 0.00 | 1.94 | 0.92 | 0.09 | 0.16 | 0.14 | 0.25 |
|  |  | medium | 6.00 | 0.00 | 1.63 | 0.02 | 0.00 | 0.00 | 1.05 | 0.04 |  |  | medium | 6.00 | 0.00 | 0.37 | 0.08 | 0.00 | 0.00 | 1.57 | 0.80 |
|  |  | high | 5.00 | 0.00 | 0.00 | 0.00 | 0.00 | 0.00 | 0.88 | 0.07 |  |  | high | 5.00 | 0.00 | 34.2 | 2.01 | 1.78 | 3.09 | 1.45 | 0.41 |
|  | syringic acid | low | 7.00 | 0.00 | 1.45 | 0.18 | 0.00 | 0.00 | 0.42 | 0.07 |  | syringic acid | low | 7.00 | 0.00 | 2.69 | 1.32 | 0.15 | 0.13 | 0.00 | 0.00 |
|  |  | medium | 6.50 | 0.00 | 1.69 | 0.16 | 0.00 | 0.00 | 4.28 | 0.14 |  |  | medium | 6.50 | 0.00 | 0.77 | 0.11 | 0.00 | 0.00 | 4.09 | 0.14 |
|  |  | high | 5.00 | 0.00 | 0.49 | 0.06 | 0.00 | 0.00 | 0.00 | 0.00 |  |  | high | 5.00 | 0.00 | 0.78 | 0.44 | 3.80 | 0.83 | 0.44 | 0.38 |
|  | vanillic acid | low | 7.00 | 0.00 | 1.78 | 0.06 | 0.00 | 0.00 | 0.28 | 0.03 |  | vanillic acid | low | 7.00 | 0.00 | 2.27 | 0.62 | 0.04 | 0.08 | 0.00 | 0.00 |
|  |  | medium | 7.00 | 0.00 | 2.29 | 0.13 | 0.15 | 0.01 | 1.83 | 0.02 |  |  | medium | 7.00 | 0.00 | 8.36 | 0.29 | 0.00 | 0.00 | 1.77 | 0.20 |
|  |  | high | 5.00 | 0.00 | 0.58 | 0.03 | 0.00 | 0.00 | 0.00 | 0.00 |  |  | high | 5.00 | 0.00 | 0.45 | 0.03 | 3.86 | 0.40 | 0.13 | 0.11 |
|  | ferulic acid | low | 7.00 | 0.00 | 4.32 | 0.50 | 0.00 | 0.00 | 0.15 | 0.04 |  | ferulic acid | low | 7.00 | 0.00 | 3.98 | 0.39 | 0.20 | 0.34 | 0.00 | 0.00 |
|  |  | medium | 6.50 | 0.00 | 7.27 | 3.36 | 0.22 | 0.03 | 0.22 | 0.39 |  |  | medium | 6.50 | 0.00 | 6.17 | 4.28 | 1.82 | 0.30 | 0.62 | 0.29 |
|  |  | high | 5.00 | 0.00 | 0.85 | 0.09 | 0.00 | 0.00 | 0.00 | 0.00 |  |  | high | 5.00 | 0.00 | 0.33 | 0.29 | 4.15 | 0.32 | 0.00 | 0.00 |
|  | coumaric acid | low | 7.00 | 0.00 | 1.86 | 0.15 | 0.08 | 0.07 | 0.36 | 0.10 |  | coumaric acid | low | 7.00 | 0.00 | 2.91 | 0.94 | 0.41 | 0.16 | 0.81 | 0.16 |
|  |  | medium | 7.00 | 0.00 | 0.91 | 0.06 | 0.25 | 0.02 | 1.61 | 0.32 |  |  | medium | 7.00 | 0.00 | 2.46 | 0.33 | 0.20 | 0.04 | 2.31 | 0.21 |
|  |  | high | 5.00 | 0.00 | 0.47 | 0.03 | 0.00 | 0.00 | 1.91 | 0.25 |  |  | high | 5.00 | 0.00 | 0.51 | 0.02 | 4.07 | 0.07 | 8.03 | 0.27 |

Table S5: Concentrations of phenylacetate (PAA), phenylpropionate (PPA), and phenylbutyrate (PBA, sum) of thermophilic control samples as well as of thermophilic samples fed with the respective lignin intermediate (gallic, syringic, vanillic, ferulic, or coumaric acid) under low, medium, and high overload conditions on day 0, 7, 14, and 28 . “PBA sum” represents the sum of the 2-PBA, 3-PBA, and 4-PBA concentrations of each variation. Results are shown in mM. Mean values are presented with the respective standard deviations (SD).

| **Day** | **Source** | **Load** | **PAA** | | **PPA** | | **PBA (sum)** | | **Day** | **Source** | **Load** | **PAA** | | **PPA** | | **PBA (sum)** | |
| --- | --- | --- | --- | --- | --- | --- | --- | --- | --- | --- | --- | --- | --- | --- | --- | --- | --- |
|  |  |  | **mean** | **SD** | **mean** | **SD** | **mean** | **SD** |  |  |  | **mean** | **SD** | **mean** | **SD** | **mean** | **SD** |
| 0 | control | control | 0.22 | 0.00 | 0.10 | 0.01 | 0.02 | 0.00 | 14 | control | control | 0.20 | 0.01 | 0.09 | 0.02 | 0.10 | 0.03 |
|  | gallic acid | low | 0.26 | 0.00 | 0.09 | 0.00 | 0.03 | 0.00 |  | gallic acid | low | 0.21 | 0.02 | 0.09 | 0.02 | 0.09 | 0.02 |
|  |  | medium | 0.29 | 0.00 | 0.07 | 0.00 | 0.02 | 0.00 |  |  | medium | 0.22 | 0.02 | 0.08 | 0.01 | 0.08 | 0.01 |
|  |  | high | 0.49 | 0.09 | 0.00 | 0.01 | 0.04 | 0.00 |  |  | high | 3.53 | 0.42 | 0.01 | 0.01 | 0.01 | 0.01 |
|  | syringic acid | low | 0.27 | 0.00 | 0.11 | 0.00 | 0.02 | 0.00 |  | syringic acid | low | 0.23 | 0.01 | 0.07 | 0.02 | 0.07 | 0.02 |
|  |  | medium | 0.40 | 0.00 | 0.14 | 0.00 | 0.01 | 0.01 |  |  | medium | 0.26 | 0.04 | 0.07 | 0.01 | 0.07 | 0.01 |
|  |  | high | 0.28 | 0.00 | 0.27 | 0.00 | 0.01 | 0.00 |  |  | high | 0.21 | 0.01 | 0.01 | 0.02 | 0.07 | 0.03 |
|  | vanillic acid | low | 0.28 | 0.01 | 0.00 | 0.00 | 0.00 | 0.00 |  | vanillic acid | low | 0.26 | 0.01 | 0.10 | 0.01 | 0.10 | 0.01 |
|  |  | medium | 0.28 | 0.00 | 0.00 | 0.00 | 0.00 | 0.00 |  |  | medium | 0.27 | 0.01 | 0.10 | 0.01 | 0.10 | 0.01 |
|  |  | high | 0.89 | 0.00 | 0.00 | 0.00 | 0.00 | 0.00 |  |  | high | 0.23 | 0.00 | 0.00 | 0.00 | 0.00 | 0.00 |
|  | ferulic acid | low | 0.22 | 0.00 | 0.24 | 0.00 | 0.01 | 0.01 |  | ferulic acid | low | 0.31 | 0.01 | 0.52 | 0.02 | 0.52 | 0.02 |
|  |  | medium | 0.27 | 0.01 | 0.00 | 0.00 | 0.03 | 0.00 |  |  | medium | 0.73 | 0.26 | 0.21 | 0.15 | 0.34 | 0.13 |
|  |  | high | 0.23 | 0.00 | 0.00 | 0.00 | 0.14 | 0.00 |  |  | high | 0.14 | 0.08 | 0.00 | 0.00 | 0.03 | 0.01 |
|  | coumaric acid | low | 0.45 | 0.00 | 0.09 | 0.00 | 0.00 | 0.00 |  | coumaric acid | low | 0.34 | 0.02 | 0.54 | 0.07 | 0.55 | 0.05 |
|  |  | medium | 0.53 | 0.01 | 0.12 | 0.00 | 0.20 | 0.00 |  |  | medium | 0.83 | 0.12 | 5.45 | 0.09 | 1.45 | 0.08 |
|  |  | high | 0.89 | 0.00 | 0.00 | 0.00 | 0.04 | 0.02 |  |  | high | 14.93 | 1.72 | 3.21 | 0.38 | 0.44 | 0.02 |
| 7 | control | control | 0.02 | 0.02 | 0.01 | 0.02 | 0.00 | 0.00 | 28 | control | control | 0.21 | 0.01 | 0.03 | 0.02 | 0.03 | 0.01 |
|  | gallic acid | low | 0.00 | 0.01 | 0.00 | 0.00 | 0.00 | 0.00 |  | gallic acid | low | 0.23 | 0.01 | 0.05 | 0.01 | 0.05 | 0.06 |
|  |  | medium | 0.00 | 0.00 | 0.00 | 0.00 | 0.00 | 0.00 |  |  | medium | 0.26 | 0.02 | 0.07 | 0.00 | 0.00 | 0.01 |
|  |  | high | 0.20 | 0.19 | 0.00 | 0.00 | 0.00 | 0.00 |  |  | high | 0.91 | 0.31 | 0.01 | 0.01 | 0.01 | 0.01 |
|  | syringic acid | low | 0.37 | 0.63 | 0.00 | 0.00 | 0.00 | 0.00 |  | syringic acid | low | 0.25 | 0.01 | 0.01 | 0.02 | 0.31 | 0.24 |
|  |  | medium | 0.00 | 0.00 | 0.01 | 0.01 | 0.00 | 0.01 |  |  | medium | 0.26 | 0.03 | 0.06 | 0.02 | 0.00 | 0.01 |
|  |  | high | 0.00 | 0.00 | 0.00 | 0.00 | 0.07 | 0.00 |  |  | high | 0.33 | 0.01 | 0.21 | 0.07 | 0.24 | 0.02 |
|  | vanillic acid | low | 0.03 | 0.01 | 0.00 | 0.00 | 0.00 | 0.00 |  | vanillic acid | low | 0.26 | 0.02 | 0.00 | 0.01 | 0.41 | 0.12 |
|  |  | medium | 0.00 | 0.00 | 0.03 | 0.02 | 0.00 | 0.00 |  |  | medium | 0.23 | 0.01 | 0.08 | 0.00 | 0.00 | 0.01 |
|  |  | high | 0.00 | 0.00 | 0.17 | 0.03 | 0.00 | 0.00 |  |  | high | 0.71 | 0.02 | 1.43 | 0.02 | 0.00 | 0.00 |
|  | ferulic acid | low | 0.15 | 0.12 | 0.08 | 0.05 | 0.00 | 0.01 |  | ferulic acid | low | 0.28 | 0.01 | 0.00 | 0.00 | 1.69 | 0.03 |
|  |  | medium | 4.61 | 4.22 | 0.19 | 0.06 | 0.07 | 0.04 |  |  | medium | 1.12 | 0.68 | 0.75 | 0.14 | 0.00 | 0.00 |
|  |  | high | 0.00 | 0.00 | 0.00 | 0.00 | 0.10 | 0.07 |  |  | high | 18.4 | 0.70 | 0.00 | 0.00 | 0.00 | 0.00 |
|  | coumaric acid | low | 2.48 | 0.07 | 0.88 | 0.07 | 0.05 | 0.04 |  | coumaric acid | low | 0.34 | 0.00 | 0.78 | 0.17 | 0.09 | 0.04 |
|  |  | medium | 2.09 | 0.15 | 1.85 | 0.15 | 0.15 | 0.13 |  |  | medium | 0.87 | 0.15 | 6.78 | 0.10 | 1.08 | 0.12 |
|  |  | high | 5.28 | 0.23 | 3.47 | 0.96 | 1.22 | 0.09 |  |  | high | 18.0 | 0.66 | 1.90 | 0.30 | 0.54 | 0.10 |

Table S6: Concentrations of benzoate, hydroxybenzoate, and hydroxy-PAA of thermophilic control samples as well as of thermophilic samples fed with the respective lignin intermediate (gallic, syringic, vanillic, ferulic, or coumaric acid) under low, medium, and high overload conditions on day 0, 7, 14, and 28 . Results are shown in mM. Mean values are presented with the respective standard deviations (SD).

| **Day** | **Source** | **Load** | **Benzoate** | | **Hydroxy-benzoate** | | **Hydroxy-PAA** | | **Day** | **Source** | **Load** | **Benzoate** | | **Hydroxy-benzoate** | | **Hydroxy-PAA** | |
| --- | --- | --- | --- | --- | --- | --- | --- | --- | --- | --- | --- | --- | --- | --- | --- | --- | --- |
|  |  |  | **mean** | **SD** | **mean** | **SD** | **mean** | **SD** |  |  |  | **mean** | **SD** | **mean** | **SD** | **mean** | **SD** |
| 0 | control | control | 0.00 | 0.00 | 0.00 | 0.00 | 0.00 | 0.00 | 14 | control | control | 0.00 | 0.00 | 0.01 | 0.01 | 0.01 | 0.01 |
|  | gallic acid | low | 0.00 | 0.00 | 0.00 | 0.00 | 0.00 | 0.00 |  | gallic acid | low | 0.00 | 0.00 | 0.01 | 0.01 | 0.00 | 0.00 |
|  |  | medium | 0.00 | 0.00 | 0.00 | 0.00 | 0.00 | 0.00 |  |  | medium | 0.00 | 0.00 | 0.00 | 0.00 | 0.13 | 0.04 |
|  |  | high | 0.00 | 0.00 | 0.00 | 0.00 | 0.00 | 0.00 |  |  | high | 0.00 | 0.00 | 0.06 | 0.05 | 0.00 | 0.00 |
|  | syringic acid | low | 0.00 | 0.00 | 0.00 | 0.00 | 0.00 | 0.00 |  | syringic acid | low | 0.00 | 0.00 | 0.03 | 0.05 | 0.00 | 0.00 |
|  |  | medium | 0.00 | 0.00 | 0.02 | 0.00 | 0.00 | 0.00 |  |  | medium | 0.00 | 0.00 | 0.00 | 0.00 | 0.00 | 0.00 |
|  |  | high | 0.00 | 0.00 | 0.07 | 0.00 | 0.00 | 0.00 |  |  | high | 0.00 | 0.00 | 0.00 | 0.00 | 0.01 | 0.02 |
|  | vanillic acid | low | 0.00 | 0.00 | 0.00 | 0.00 | 0.00 | 0.00 |  | vanillic acid | low | 0.00 | 0.00 | 0.04 | 0.04 | 0.06 | 0.05 |
|  |  | medium | 0.00 | 0.00 | 0.00 | 0.00 | 0.00 | 0.00 |  |  | medium | 0.00 | 0.00 | 0.00 | 0.00 | 2.06 | 0.18 |
|  |  | high | 0.00 | 0.00 | 0.00 | 0.00 | 0.00 | 0.00 |  |  | high | 0.00 | 0.00 | 0.10 | 0.02 | 0.00 | 0.00 |
|  | ferulic acid | low | 0.00 | 0.00 | 0.00 | 0.00 | 0.00 | 0.00 |  | ferulic acid | low | 0.00 | 0.00 | 0.00 | 0.00 | 0.00 | 0.00 |
|  |  | medium | 0.00 | 0.00 | 0.00 | 0.00 | 0.00 | 0.00 |  |  | medium | 0.00 | 0.00 | 0.00 | 0.00 | 0.15 | 0.01 |
|  |  | high | 0.00 | 0.00 | 0.00 | 0.00 | 0.00 | 0.00 |  |  | high | 0.00 | 0.00 | 0.00 | 0.00 | 0.02 | 0.02 |
|  | coumaric acid | low | 0.00 | 0.00 | 0.00 | 0.00 | 0.00 | 0.00 |  | coumaric acid | low | 0.00 | 0.00 | 0.00 | 0.00 | 0.00 | 0.00 |
|  |  | medium | 0.00 | 0.00 | 0.00 | 0.00 | 0.00 | 0.00 |  |  | medium | 0.00 | 0.00 | 0.00 | 0.00 | 0.00 | 0.00 |
|  |  | high | 0.00 | 0.00 | 0.00 | 0.00 | 0.00 | 0.00 |  |  | high | 0.00 | 0.00 | 0.00 | 0.00 | 0.00 | 0.00 |
| 7 | control | control | 0.00 | 0.00 | 0.00 | 0.00 | 0.00 | 0.00 | 28 | control | control | 0.00 | 0.00 | 0.00 | 0.00 | 0.00 | 0.00 |
|  | gallic acid | low | 0.00 | 0.00 | 0.00 | 0.00 | 0.00 | 0.00 |  | gallic acid | low | 0.01 | 0.00 | 0.00 | 0.00 | 0.00 | 0.00 |
|  |  | medium | 0.00 | 0.00 | 0.00 | 0.00 | 0.00 | 0.00 |  |  | medium | 0.01 | 0.00 | 0.00 | 0.00 | 0.17 | 0.05 |
|  |  | high | 0.00 | 0.00 | 0.00 | 0.00 | 0.00 | 0.00 |  |  | high | 0.00 | 0.00 | 0.10 | 0.00 | 0.00 | 0.00 |
|  | syringic acid | low | 0.00 | 0.00 | 0.00 | 0.00 | 0.00 | 0.00 |  | syringic acid | low | 0.00 | 0.00 | 0.00 | 0.00 | 0.00 | 0.00 |
|  |  | medium | 0.00 | 0.00 | 0.00 | 0.00 | 0.00 | 0.00 |  |  | medium | 0.01 | 0.00 | 0.00 | 0.00 | 0.00 | 0.00 |
|  |  | high | 0.00 | 0.00 | 0.18 | 0.00 | 0.18 | 0.00 |  |  | high | 0.00 | 0.00 | 0.00 | 0.00 | 0.00 | 0.00 |
|  | vanillic acid | low | 0.00 | 0.00 | 0.00 | 0.00 | 0.00 | 0.00 |  | vanillic acid | low | 0.00 | 0.00 | 0.00 | 0.00 | 0.00 | 0.00 |
|  |  | medium | 0.00 | 0.00 | 0.00 | 0.00 | 0.00 | 0.00 |  |  | medium | 0.01 | 0.00 | 0.00 | 0.00 | 0.00 | 0.00 |
|  |  | high | 0.00 | 0.00 | 0.07 | 0.01 | 0.07 | 0.01 |  |  | high | 0.00 | 0.00 | 0.00 | 0.00 | 0.00 | 0.00 |
|  | ferulic acid | low | 0.00 | 0.00 | 0.00 | 0.00 | 0.00 | 0.00 |  | ferulic acid | low | 0.00 | 0.00 | 0.00 | 0.00 | 0.00 | 0.00 |
|  |  | medium | 0.00 | 0.00 | 0.34 | 0.37 | 0.34 | 0.37 |  |  | medium | 0.11 | 0.02 | 0.00 | 0.00 | 0.16 | 0.04 |
|  |  | high | 0.00 | 0.00 | 0.00 | 0.00 | 0.00 | 0.00 |  |  | high | 0.00 | 0.00 | 0.00 | 0.00 | 0.01 | 0.00 |
|  | coumaric acid | low | 0.00 | 0.00 | 0.00 | 0.00 | 0.00 | 0.00 |  | coumaric acid | low | 0.11 | 0.02 | 0.00 | 0.00 | 0.00 | 0.00 |
|  |  | medium | 0.00 | 0.00 | 0.02 | 0.02 | 0.02 | 0.02 |  |  | medium | 0.00 | 0.00 | 0.00 | 0.00 | 0.00 | 0.00 |
|  |  | high | 0.00 | 0.00 | 0.02 | 0.01 | 0.02 | 0.01 |  |  | high | 0.00 | 0.00 | 0.00 | 0.00 | 0.01 | 0.00 |

Figure S1a: Mean sequence proportions [%] of mesophilic genera of all control and gallic (left), syringic (middle), and vanillic acid (right) samples (White’s non-parametric t-test, p<0.05, B-H adjusted, effect size >1).

Figure S1b: Mean sequence proportions [%] of mesophilic genera of all control and ferulic acid (left) and of all control and coumaric acid (right) samples (White’s non-parametric t-test, p<0.05, B-H adjusted, effect size >1).

**Figure S2a**: Interactive visualisation of mesophilic taxa of the control as well of the gallic-, syringic-, vanillic-, ferulic-, and coumaric acid samples at low (LCL), medium (MCL), and high (HCL) overload conditions on day 0 (control) and on day 14 (all samples). The link was generated via the internet browser Mozilla Firefox™.

**Figure S2b**: Interactive visualisation of mesophilic taxa of the control as well of the gallic-, syringic-, vanillic-, ferulic-, and coumaric acid samples at low (LCL), medium (MCL), and high (HCL) overload conditions on day 0 (control) and on day 28 (all samples). The link was generated via the internet browser Mozilla Firefox™.

**Figure S3:** The Kyoto Encyclopedia of Genes and Genomes (KEGG) orthology counts of general pathways and of pathways relevant for anaerobic degradation of aromatic compounds of all mesophilic samples of day 28. Kruskal-Wallis (KW-H) analyses are included. The markers represent the medians, the boxes show the upper to lower quartiles of each median.

**Figure S4a**: Mean sequence proportions [%] of thermophilic genera of all control and gallic (left), syringic (middle), and vanillic acid (right) samples (White’s non-parametric t-test, p<0.05, B-H adjusted, effect size >1).

**Figure S4b**: Mean sequence proportions [%] of thermophilic genera of all control and ferulic acid (left) and of all control and coumaric acid (right) samples (White’s non-parametric t-test, p<0.05, B-H adjusted, effect size >1).

**Figure S5a**: Interactive visualisation of thermophilic taxa of the control as well of the gallic-, syringic-, vanillic-, ferulic-, and coumaric acid samples at low (LCL), medium (MCL), and high (HCL) overload conditions on day 0 (control) and on day 14 (all samples). The link was generated via the internet browser Mozilla Firefox™.

**Figure S5b**: Interactive visualisation of thermophilic taxa of the control as well of the gallic-, syringic-, vanillic-, ferulic-, and coumaric acid samples at low (LCL), medium (MCL), and high (HCL) overload conditions on day 0 (control) and on day 28 (all samples). The link was generated via the internet browser Mozilla Firefox™.

**Figure S6**: The Kyoto Encyclopedia of Genes and Genomes (KEGG) orthology counts of general pathways and of pathways relevant for anaerobic degradation of aromatic compounds of all thermophilic samples of 28. Kruskal-Wallis (KW-H) analyses are included. The markers represent the medians, the boxes show the upper to lower quartiles of each median.

**Figure S7**: Shannon diversity index for meso- and thermophilic samples fed with gallic (1st column), syringic (2nd column), vanillic (3rd column), ferulic (4th column), and coumaric acid (5th column).

**Figure S8a**: Neighbour joining clustering with KEGG enzyme results of thermophilic samples on day 28. The Bray-Curtis similarity index was applied.

**Figure S8b**: Heatmap (blue to red) using KEGG orthology counts of the most abundant and most differently expressed enzymes (orthology count sum ≥ 10,000 and standard deviation ≥ 500) of thermophilic controls and HCL samples of day 28. Bottom, horizontal: expressed enzymes labelled after the KEGG orthology database.

**Figure S8c**: KEGG orthology counts per samples of the enzyme subunits K00282 (glycin dehydrogenase (gcvP) subunit 1) and K00283 (gcvP subunit 2) as well of K01990 (ABC-2 type transport system ATP-binding protein) of thermophilic controls and HCL samples of day 28. Marker points and boxes show medians and lower - upper quartiles (25% - 75%), respectively

**Figure S1a**

**Figure S1b**

**Figure S2a**

**Figure S2b**


Figure S3

Figure S4a

Figure S4b

**Figure S5a**

**Figure S5b**


**Figure S6**

**Figure S7**


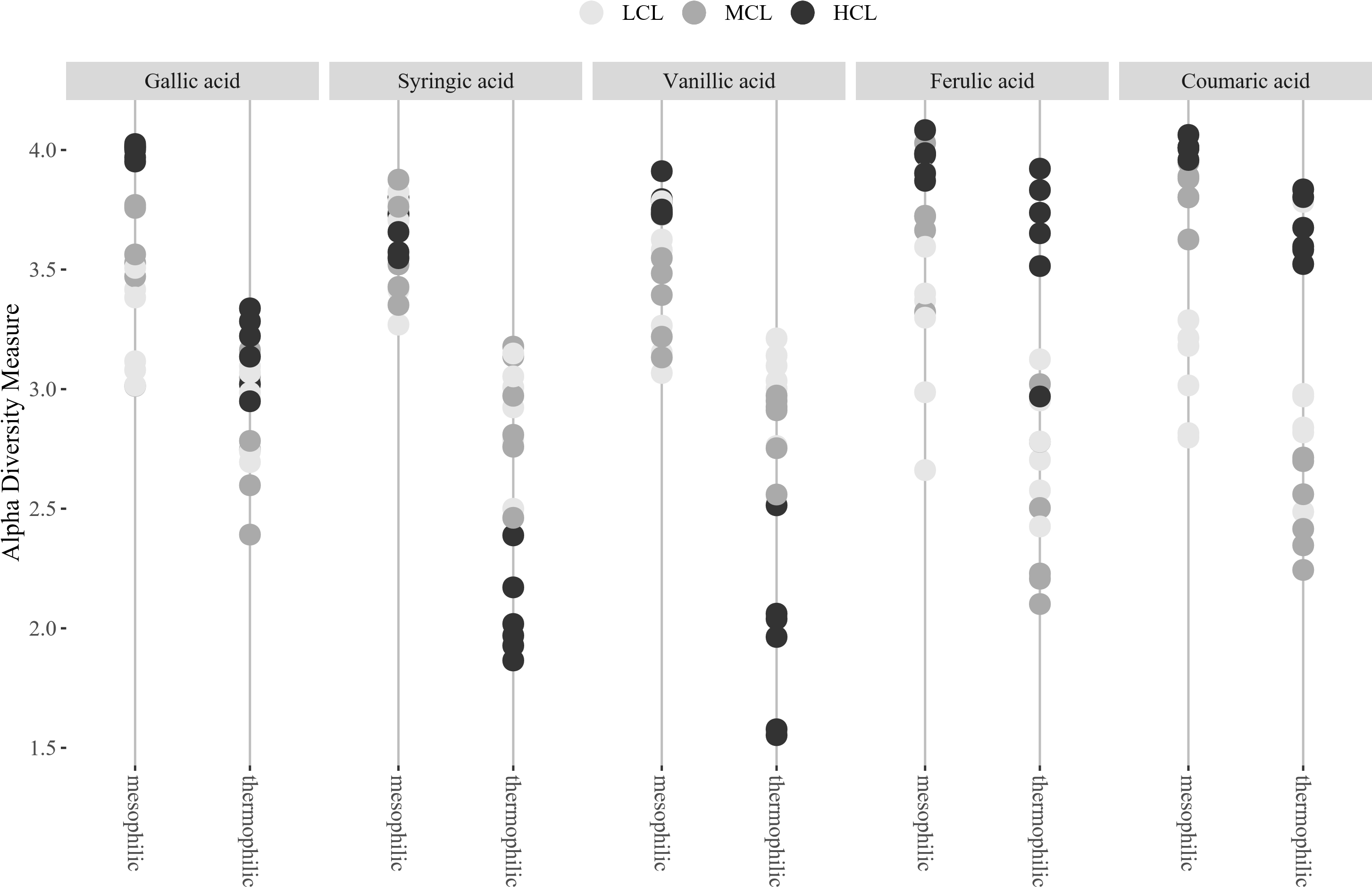


**Figure S8a**


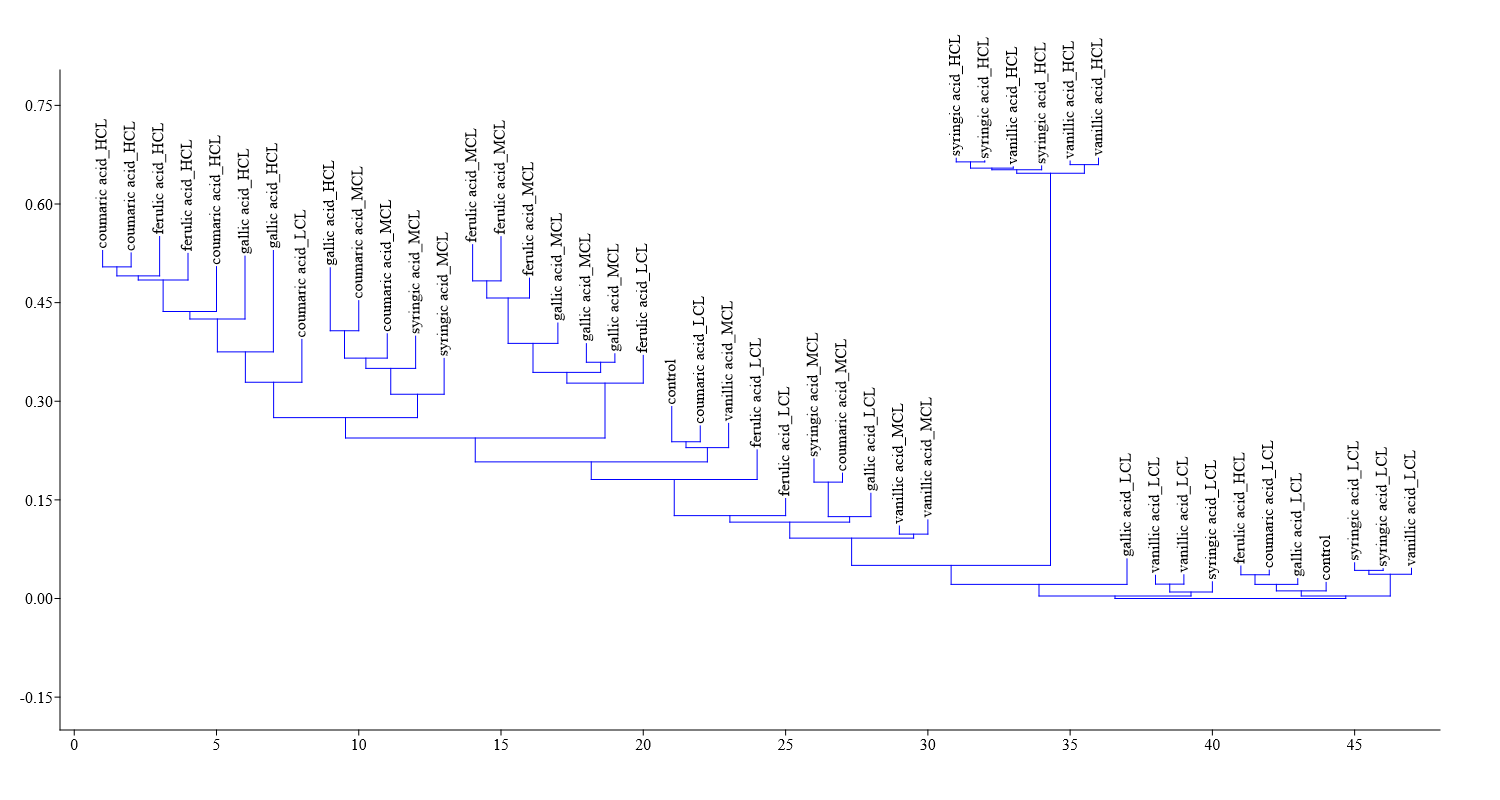


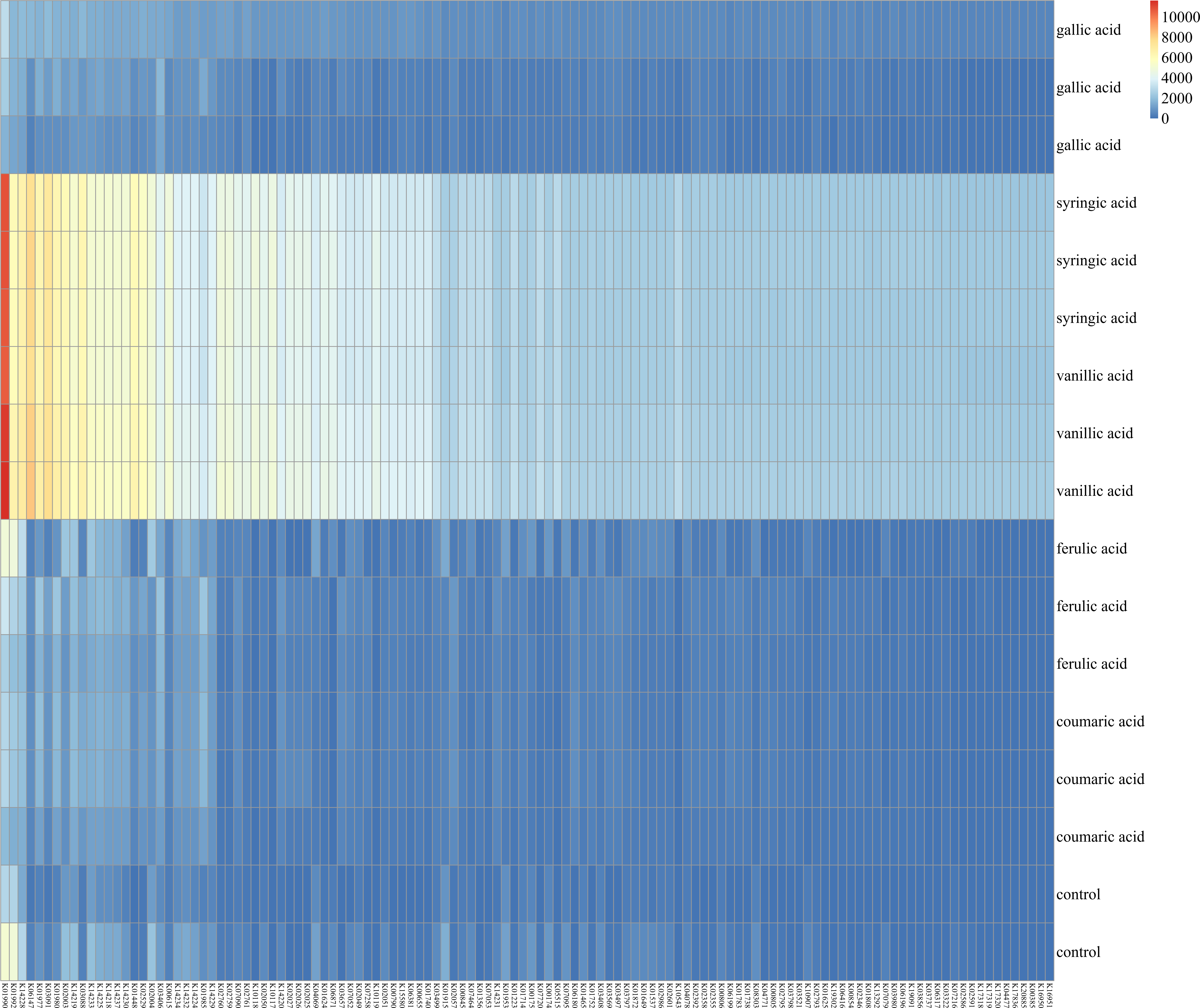
**Figure S8b**

**Figure S8c**

1. Supplementary Discussion

**Text S1:** KEGG analyses showed that K01990 (ABC-2 type transport system ATP-binding protein) was highly expressed in thermophilic syringic and vanillic HCL samples (Fig. S8b and S8c). The high orthology counts for K01990 (as well as for K00282 and K00283) can be explained by the fact that i) the protein was pre-dominantly associated with *Thermoanaerobacterium thermosaccharolyticum* and ii) by the dominance of *Thermoanaerobacterium* spp. in syringic and vanillic HCL samples (Fig. S5). In general, ATP- binding cassette (ABC) transporters are multi-subunit membrane pumps needed to energise the vectorial transfer of various substrates through membranes via ATP binding and hydrolysation [1]. While *Thermoanaerobacterium* spp. was primarily associated with K01990 in syringic and vanillic HCL samples, the hydrogenotrophic methanogens *Methanoculleus bourgensis* and M. sp. MAB 1 were responsible for high K01990 expressions in all other samples. This are interesting first insights in the substrate transport mechanisms of anaerobic microbial consortia; however, deeper knowledge is still pending, especially when considering the role of substrate transfer during syntrophic co-operations in anaerobic (methanogenic) systems [2].

1. References

1. Rice AJ, Park A, Pinkett HW. Diversity in ABC transporters: Type I, II and III importers. Crit Rev Biochem Mol Biol. 2014;49:426–37. doi:10.3109/10409238.2014.953626.

2. Westerholm M, Moestedt J, Schnürer A. Biogas production through syntrophic acetate oxidation and deliberate operating strategies for improved digester performance. Applied Energy. 2016;179:124–35. doi:10.1016/j.apenergy.2016.06.061.
